# Resistance to the CHK1 inhibitor prexasertib involves functionally distinct CHK1 activities in BRCA wild-type ovarian cancer

**DOI:** 10.1038/s41388-020-1383-4

**Published:** 2020-07-09

**Authors:** Jayakumar Nair, Tzu-Ting Huang, Junko Murai, Brittany Haynes, Patricia S. Steeg, Yves Pommier, Jung-Min Lee

**Affiliations:** 1 Women’s Malignancies Branch, National Institutes of Health, Bethesda, 20892 MD USA; 2 Developmental Therapeutics Branch and Laboratory of Molecular Pharmacology, Center for Cancer Research, National Cancer Institute, National Institutes of Health, Bethesda, 20892 MD USA

**Keywords:** Ovarian cancer, Ovarian cancer, Target identification, Target identification

## Abstract

High grade serous ovarian cancer (HGSOC) is a fatal gynecologic malignancy in the U.S. with limited treatment options. New therapeutic strategies include targeting of the cell cycle checkpoints, e.g., ATR and CHK1. We recently reported a promising clinical activity of the CHK1 inhibitor (CHK1i) prexasertib monotherapy in *BRCA* wild-type (BRCAwt) HGSOC patients. In this study, biopsies of treated patients and cell line models were used to investigate possible mechanisms of resistance to CHK1i. We report that BRCAwt HGSOC develops resistance to prexasertib monotherapy via a prolonged G2 delay induced by lower CDK1/CyclinB1 activity, thus preventing cells from mitotic catastrophe and cell death. On the other hand, we noted CHK1’s regulation on RAD51-mediated homologous recombination (HR) repair was not altered in CHK1i-resistant cells. Therefore, CHK1i sensitizes CHK1i-resistant cells to DNA damaging agents such as gemcitabine or hydroxyurea by inhibition of HR. In summary, our results demonstrate new mechanistic insights of functionally distinct CHK1 activities and highlight a potential combination treatment approach to overcome CHK1i resistance in BRCAwt HGSOC.

## Introduction

High grade serous ovarian cancer (HGSOC) is the most lethal gynecologic malignancy in the United States [1]. Recurrence is nearly universal after initial platinum-based chemotherapy, leading to incurable disease and limited treatment options [2]. Approximately 25% of HGSOC are deficient in homologous recombination (HR) DNA double-strand break (DSB) repair due to *BRCA1* and *BRCA2* germline or somatic mutations [3, 4] sensitizing them to DNA damaging agents and PARP inhibitors (PARPis). PARPis have led to a new treatment paradigm in ovarian cancer. However, a majority of patients have no *BRCA* mutations and derive limited clinical benefit from PARPi monotherapy. Hence, a critical need remains for new effective therapeutic strategies for HGSOC without *BRCA* mutations and understanding resistance mechanisms associated with such treatments.

A strategy to modulate DNA repair response in *BRCA* wild-type (BRCAwt) HGSOC is to interfere with cell cycle checkpoint signaling, critical for coordination between DNA damage response and cell cycle control. Due to universal p53 dysfunction and the consequent G1 checkpoint defect, HGSOC cells depend on ataxia telangiectasia and Rad3-related (ATR)/cell cycle checkpoint kinase1 (CHK1)-mediated G2/M cell cycle arrest for DNA repair [5]. CHK1 also plays important roles in stabilizing replication forks by regulating origin firing [6], and facilitating nuclear translocation and interactions between BRCA2 and RAD51, essential for HR [7]. Therefore, targeting of cell cycle checkpoints is a promising therapeutic strategy to augment replication stress while attenuating DNA repair responses.

We recently reported clinical activity of the CHK1 inhibitor (CHK1i) prexasertib (Prex) in recurrent BRCAwt HGSOC where half of heavily pretreated patients attained clinical benefit [8]. While exciting, half of patients did not derive clinical benefit and mechanisms of resistance to CHK1i remain unknown. In the current study, we used tissue biopsies from HGSOC patients for subsequent transcriptome analysis and report the enrichment of genes of single-stranded DNA break (SSB) repair pathways in both CHK1i-resistant HGSOC cell lines and clinical samples.

For further mechanistic studies, we developed Prex-resistant (PrexR) cell lines and found that PrexR HGSOC cells have a large CyclinB1-negative G2 population and lower CDK1 activity, while parental cells demonstrate a CyclinB1-positive G2 population at baseline. Moreover, CHK1i-resistant cells did not accumulate in S phase upon treatment of Prex, instead showed a delayed progression at G2 phase due to lower CDK1/CyclinB1 activity, thus avoiding early mitotic entry and mitotic catastrophe. The consequent resistance to unscheduled mitotic entry and a sustained SSB repair process are therefore major contributory factors to Prex resistance when Prex was used as monotherapy in BRCAwt HGSOC. On the other hand, we found continued inhibition of RAD51-mediated HR by Prex in PrexR cells thus making them vulnerable to DNA DSB damaging drugs such as gemcitabine or hydroxyurea (HU). Overall, our data provide novel insights into the two functionally distinct CHK1 activities. First, the regulation of G2/M checkpoint is primarily responsible for CHK1i-induced toxicity. Secondly, the HR regulatory activity plays an important role in combination therapy with DNA damaging agents thus highlighting the combination treatment strategies to overcome CHK1i resistance.

## Results

### Development and characterization of CHK1i-resistant HGSOC cell lines

**Fig. 1:**
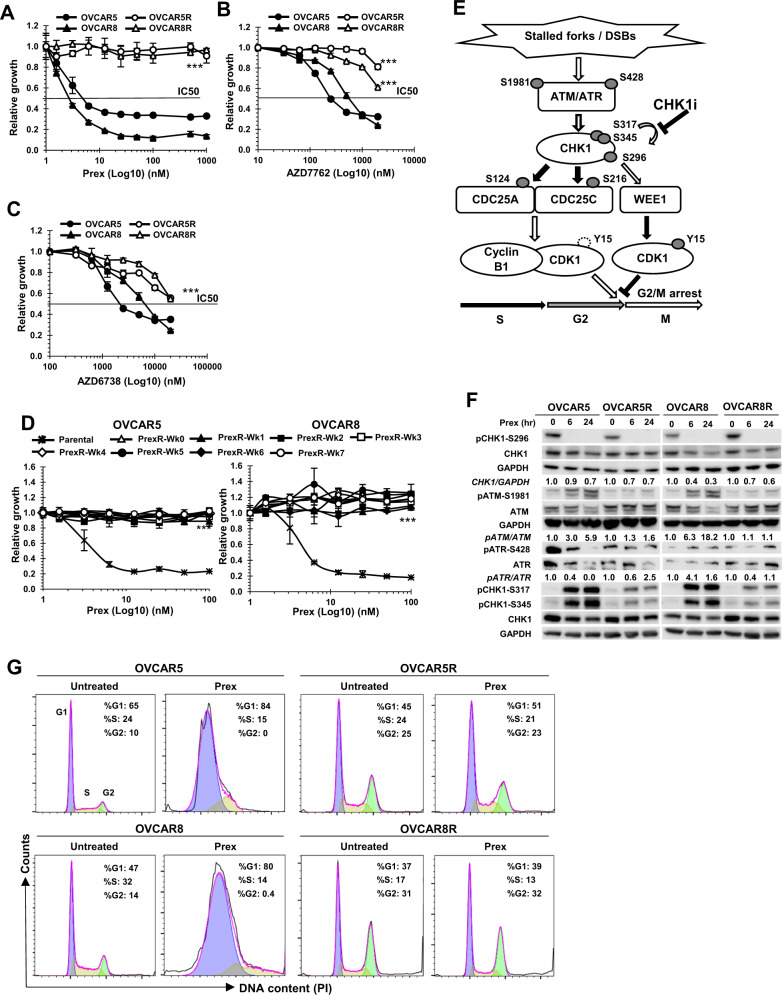
Acquired resistance to CHK1 inhibition in BRCAwt HGSOC cell lines involves a persistent G2 delay. **a**–**c** Parental cells (OVCAR5 and OVCAR8) and Prex-resistant cell lines (OVCAR5R and OVCAR8R) were treated with CHK1i Prex (0–1 µM) (**a**), another CHK1i AZD7762 (0–2 µM) (**b**), or an ATR inhibitor AZD6738 (0–20 µM) (**c**) for 48 h. The growth rates were determined by XTT assay. **d** Prex-resistant (PrexR) cell lines OVCAR5R and OVCAR8R were cultured without Prex for 7 weeks, the growth rates at indicated weeks (Wk) were measured by XTT assay. Parental cells are included as controls. **e** ATR and ATM phosphorylate CHK1 at S345 and S317 in response to DNA damage or replication stress, and subsequently induce its autophosphorylation at S296 for full activation. Activated CHK1 then phosphorylates its substrates CDC25A and CDC25C, which are essential for maintaining the dephosphorylated state of active CDK1. Accumulation of inactive CDK1 phosphorylated by WEE1 kinase at Y15 induces G2 arrest, allowing time for DNA repair prior to mitotic entry. CHK1i Prex abrogates this process, resulting in early mitotic entry with unrepaired DNA damage, leading to replication catastrophe and cell death. Open arrows indicate activating events, while shaded arrows indicate inhibitory ones. **f** Immunoblotting of pCHK1-S296, pATM-S1981, pATR-S428, pCHK1-S317, pCHK1-S345, and their respective total proteins in cells treated with Prex (20 nM) for 6 and 24 h. Densitometric values of CHK1 normalized to GAPDH and pATM or pATR normalized to ATM or ATR, relative to untreated are shown. **g** Cell cycle analysis on parental and PrexR cells treated with or without Prex (20 nM) for 24 h. The color-coded G1, S and G2 peaks are indicated in the first panel. All experiments were repeated at least thrice. Data are shown as mean ± SD. ***P < 0.001.

IC50 values for CHK1i Prex were determined to be 7.5 and 5.4 nM in OVCAR5 and OVCAR8, respectively (Fig. 1a), while IC50s were not reached for PrexR cells despite increasing concentrations up to 3 µM. PrexR cells were also cross-resistant to another CHK1i and an ATR inhibitor. IC50 values of CHK1i AZD7762 were 6 and 2.6 µM for OVCAR5R and OVCAR8R, compared with 0.4 and 0.7 µM for their respective parental lines (Fig. 1b). IC50 values of the ATR inhibitor AZD6738 were 22.4 and 22.3 µM for OVCAR5R and OVCAR8R, while they were 2.2 and 7.2 µM for the respective parent cell lines (Fig. 1c, *P* < 0.001 for both). Growth assays (XTT) on PrexR cells were performed weekly after removing CHK1i for up to 7 weeks (Fig. 1d) and confirmed sustained resistance to CHK1i (20 nM). Clonogenic assays further confirmed resistance to CHK1i at week 7 of Prex withdrawal (Supplementary Fig. 1a). Growth curve experiments with untreated cells measured over 10 days at 24 h intervals, showed longer generation times (GT) for OVCAR5R (32 h vs. 27 h for OVCAR5), while they were relatively unchanged for OVCAR8R (25 h vs. 24 h for OVCAR8) (Supplementary Fig. 1b).

Under normal culture conditions, active CDC25 phosphatases dephosphorylate and activate CDK1/CyclinB1 complex once cells enter the G2 phase, allowing transition to mitosis [9]. But, following DNA damage or replication stress, G2 delay is necessary for DNA repair. CHK1 is phosphorylated by ATR at S317 and S345 [9], and to a lesser extent by ATM on S317 [10], a prerequisite for autophosphorylation at S296 for full activation of CHK1 (Fig. 1e) [11]. Activated CHK1 then phosphorylates and inhibits the CDC25 phosphatases, enhances CDK1 phosphorylation by Wee1 kinase, thus causing G2 arrest (Fig. 1e) [11, 12]. First, we assessed the CHK1 activation by immunoblotting to exclude a possibility of CHK1 upregulation in PrexR cells. Increased phosphorylation of CHK1 following CHK1i was observed on S345 and S317 in both parental cells and PrexR cells (Fig. 1f). However, CHK1i inhibited S296 autophosphorylation of CHK1 in both cells (Fig. 1f) [13, 14], suggesting that CHK1 activity remains inhibited by CHK1i; thus drug efflux is unlikely a major mechanism of resistance. A decrease in total CHK1 levels was also observed in both parental and PrexR cells as early as 6 h after CHK1i (Fig. 1f). Based on these observations, we hypothesized that the primary mechanism of resistance to CHK1i would involve CHK1-downstream cell cycle regulators, such as CDC25 or CDK/Cyclin complexes rather than deregulation of CHK1 or upstream proteins, e.g., ATM or ATR.

### CHK1i resistance involves enforced delay at G2 phase of cell cycle

We next investigated the role of cell cycle regulation in CHK1i resistance. CHK1i causes cells to bypass G2 checkpoint and enter early mitosis, therefore leading to further sensitivity to DNA damaging agents such as cisplatin [11, 15], carboplatin [16], gemcitabine [14], and PARPis [7, 17]. In cell cycle analysis, PrexR cells showed a marked increase (>2-fold) in G2 population at baseline compared with the parental cells (25–31% in PrexR cells vs. 10–14% in parental cells) (Fig. 1g). Treatment of parental cells with 20 nM of Prex over 48 h resulted in the accumulation of cells at the S phase (Fig. 1g, left), consistent with a previous report [13]. In contrast, PrexR cells continued to show persistent delay at G2 phase (Fig. 1g, right) despite CHK1i treatment, suggesting CHK1 inhibition in PrexR cells does not mitigate the G2 delay before mitotic entry.

### CyclinB1, a key regulator of G2 checkpoint arrest in CHK1i resistance

**Fig. 2:**
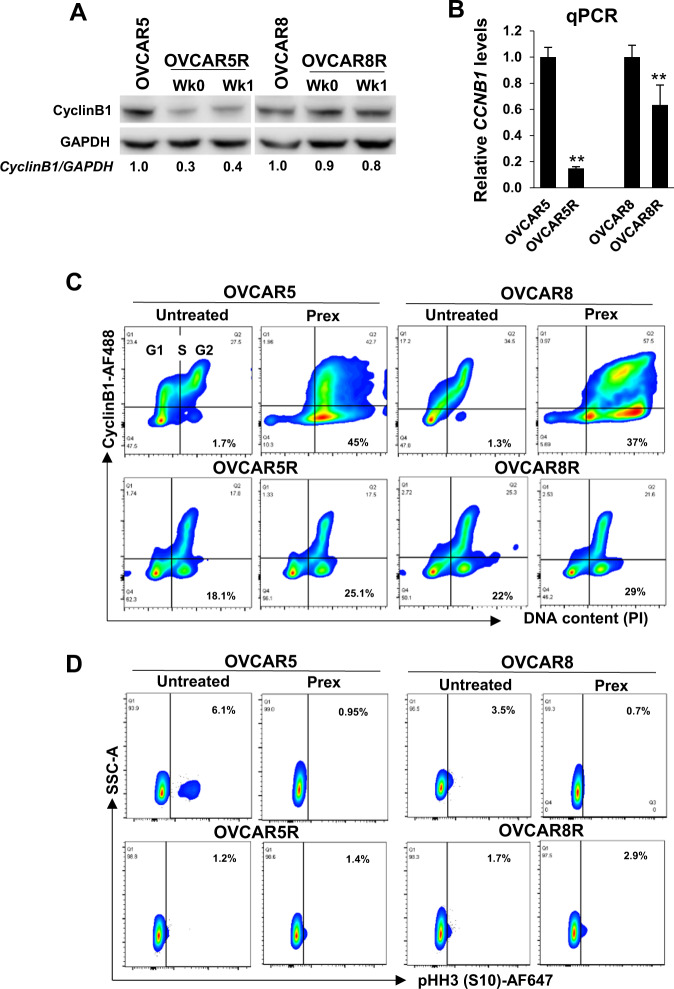
CHK1i-resistant cells show reduced CyclinB1 expression. **a** Western blot analysis of CyclinB1 in cells routinely cultured with CHK1i, Prex (1 µM) (Wk0) or following Prex withdrawal for a week (Wk1). Densitometric values of CyclinB1 relative to GAPDH are shown. **b** qPCR of *CCNB1* performed on total RNA extracted from parental or PrexR cells (cultured for 4 days without Prex). **c** Flow cytometric analysis of cells stained for CyclinB1 on the log *Y*-axis against DNA content (PI) on the linear *X*-axis. The G1, S, and G2 cell cycle phases are marked in the first plot, while the percentage of CyclinB1-negative cells are shown in bold on the right bottom panels. **d** Cell populations in (**c**) were gated on G2 and cells that stained for mitotic marker pHH3-S10 were visualized on the log *X*-axis against the side scatter channel (SSC) on the linear *Y*-axis. Numbers in bold indicate pHH3-S10 positive G2 population. All experiments were performed at least thrice. Data are shown as mean ± SD. **P < 0.01; ***P < 0.001.

To induce G2 arrest, activated CHK1 negatively regulates the phosphatase-mediated activation of CDK1/CyclinB1 by inhibiting CDC25 phosphatases [11, 12]. Levels of CyclinB1 rise through G1 and S phases and peak in the G2 phase in order to form the complex with CDK1, a prerequisite for mitotic entry [18]. We therefore performed immunoblotting and cell cycle analyses to evaluate the CDK1/CyclinB1 complex. We observed substantially lower CyclinB1 levels in OVCAR5R cells at baseline despite significant portions of PrexR cells being in the G2 phase (Fig. 2a). OVCAR8R cells also showed a modest decrease in CyclinB1 levels (Fig. 2a). These low CyclinB1 protein levels were regulated at the transcription level (Fig. 2b).

Cell cycle analysis further confirmed that high CyclinB1 expression in parental cell lines, in mostly G2 cells as expected (94–96%), whereas <50% of PrexR G2 cells did (Fig. 2c). The CyclinB1-negative G2 subset of PrexR cells was largely unaffected by Prex. Within the G2 peak subset, the mitotic population, evidenced by a mitotic marker pHH3-S10 positivity, was >5-fold and >2-fold higher in OVCAR5 and OVCAR8 compared with their respective resistant cell lines (Fig. 2d) indicating a less mitotically active G2 population in PrexR cells at baseline. Also, Prex treatment substantially reduced a mitotic population in parental cell lines while no significant effect was made in PrexR cells (Fig. 2d). These data support our hypothesis that an atypical G2 subset with lower CyclinB1 levels contribute to a significant G2 delay that is unaffected by Prex and is a contributory factor to CHK1i resistance.

### Low CDK1/CyclinB1 activity contributes to CHK1i resistance

**Fig. 3:**
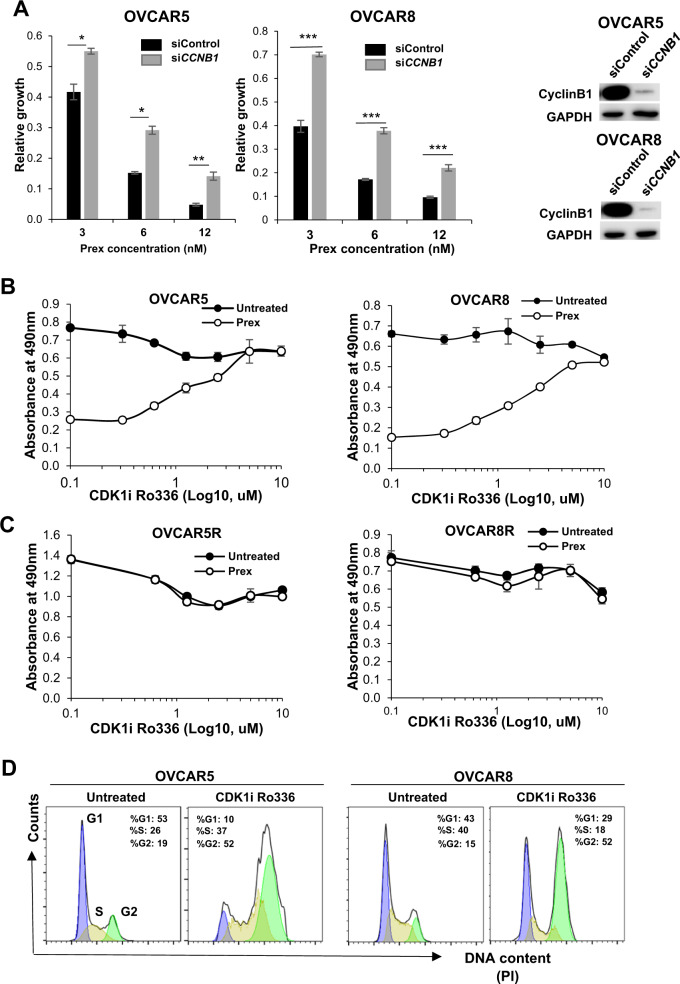
Low levels of CyclinB1 and CDK1 activity contribute to CHK1i resistance. **a** Cells transfected with either control siRNA (siControl) or CCNB1 specific siRNA (si*CCNB1*) were harvested after 48 h and used for XTT growth assays against Prex (3, 6, and 12 nM) for an additional 48 h**. b** XTT growth assay on parental and PrexR cells after 48 h of cell culture against a gradient of the CDK1 inhibitor (CDK1i) Ro336 (0–10 µM) with or without Prex (20 nM). **c** PrexR cells were similarly treated as in (**b**). **d** Cell cycle analysis of parental cells treated with Ro336 (2.5 µM). All experiments were repeated at least thrice and representative figures are shown. Data are shown as mean ± SD. *P < 0.05; **P < 0.01; ***P < 0.001.

To validate the role of CyclinB1 in our model, OVCAR5 and OVCAR8 cells were transfected with siRNA specific for *CCNB1* along with a nonspecific scrambled siRNA as control. Gene silencing resulted in undetectable levels of *CCNB1* and increased resistance to CHK1i (Fig. 3a). We then used leptomycin B, a drug blocking extranuclear export of CyclinB1 (Supplementary Fig. 2a) to investigate the effects of enforced nuclear accumulation of CyclinB1 on sensitization to Prex in both parental and PrexR cells. Both parental and PrexR cells appeared to have similar sensitivity to leptomycin B itself without additional sensitization to Prex (Supplementary Fig. 2b).

To further evaluate whether overexpression of CyclinB1 could resensitize PrexR cells to CHK1i, we transiently expressed a CyclinB1-GFP construct [19] pCMX-CCNB1GFP (pCCNB1) in both parental and PrexR cells. Growth assays over 48 h showed lower viability in cells transiently transfected with pCCNB1 with no significant sensitization to Prex (Supplementary Fig. 2c), as similarly shown with leptomycin B treatment (Supplementary Fig. 2b). We also observed that PrexR cells were more sensitive to pCCNB1 transfection relative to mock control than their parental counterparts (Supplementary Fig. 2c). Flow cytometric analysis further confirmed substantially higher apoptotic populations in both parental and PrexR cells transfected with pCCNB1 compared to mock controls (Supplementary Fig. 2d). These findings indicate that pCCNB1 transfection alone while being toxic itself, does not increase Prex-induced apoptosis in both parental and PrexR cells.

Moreover, immunofluorescent microscopic analysis showed that cell death in pCCNB1 transfected PrexR cells (green cells, Supplementary Fig. 2e) was not associated with DNA DSB, as indicated by the absence of γH2AX nuclear foci in GFP positive cells (Supplementary Fig. 2e). Overall, these results suggest that CyclinB1 overexpression alone does not reverse resistance to CHK1i in PrexR cells. This finding thus led us to explore whether a largely CyclinB1-negative G2 population in PrexR cells would also contribute to a lower nuclear CDK1 activity, which is critical for G2 delay.

To measure nuclear CDK1 activity in PrexR cells, we performed enzyme activity assays with Histone H1 and nuclear CDK1/CyclinB1 immunoprecipitates given CDK1 phosphorylates its substrate Histone H1 at T154 (pHH1-T154) for activation [20]. We found relatively lower levels of pHH1-T154 in PrexR cells compared with parental cells (Supplementary Fig. 3a). Also, immunoblotting analysis showed that inactive CDK1 levels, as measured by CDK1-Y15, was unaffected by CHK1i treatment in PrexR cells (<10% loss) while a substantial decrease of CDK1-Y15 was observed in parental OVCAR5 and OVCAR8 (70% and 50%, respectively) (Supplementary Fig. 3b). To further confirm this, we treated parental cells with increasing concentrations of a CDK1-specific inhibitor Ro336. Resistance to CHK1i increased linearly to the concentrations of Ro336 in parental lines (Fig. 3b), with no significant toxicity observed up to 10 µM of Ro336 after which marked loss in viability occurred (not shown) while showing no effect on Prex resistance in PrexR cells (Fig. 3c). Moreover, flow cytometric profiles of parental cells treated with Ro336 showed >2-fold increase in G2 population (Fig. 3d) as observed in untreated PrexR cells earlier in Fig. 1g. Together, these findings further indicate that significantly lower CDK1 activity in PrexR cells coupled with an extended G2 delay is necessary to sustain resistance to CHK1i in vitro.

### CHK1’s control over RAD51-mediated HR remains unaffected by CHK1i resistance

**Fig. 4:**
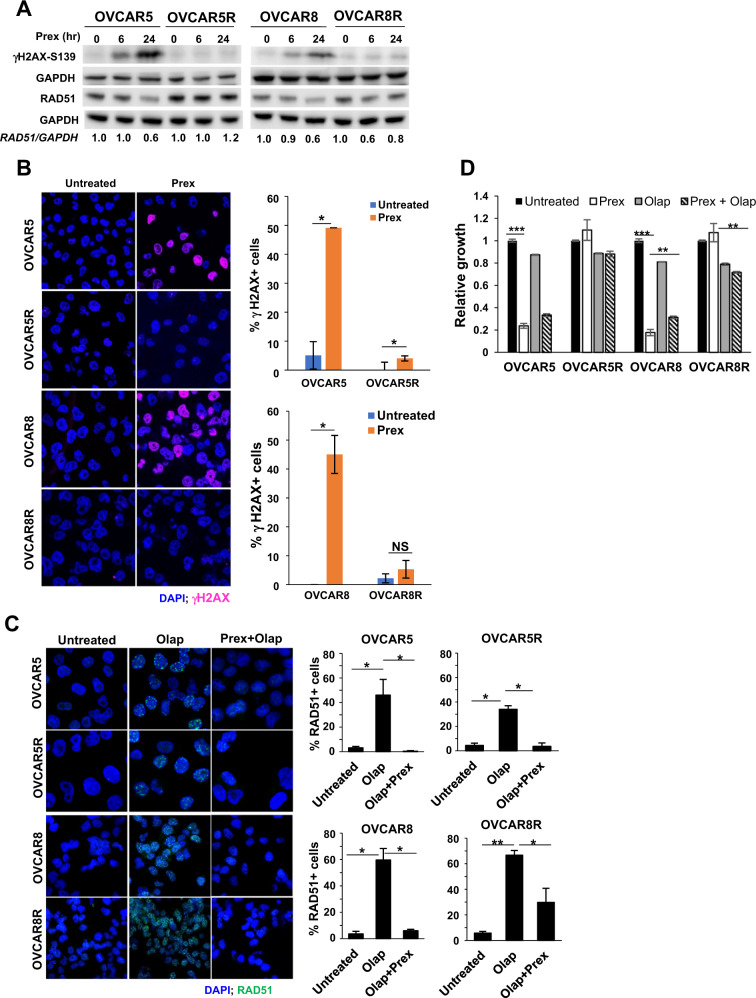
Effects of CHK1i on DNA damage and CHK1 activation. **a** Immunoblotting analysis of a DNA damage marker γH2AX and an HR marker RAD51 were performed in cells treated with Prex (20 nM) for 6 and 24 h. Densitometric quantifications of RAD51 normalized with GAPDH and relative ratios are shown. **b** Parental and PrexR cells were cultured on coverslips overnight with or without Prex (20 nM) and then fixed and stained with antibodies against γH2AX (pink) and nuclear stain DAPI (blue). Cells with >5 γH2AX foci were counted as γH2AX-positive (γH2AX+) cells. Percentage of γH2AX+ cells are plotted on the right. **c** Immunofluorescence staining of parental and PrexR cells for RAD51 foci (green) induced by PARPi olaparib (Olap) (20 µM) with or without Prex (10 nM). Nuclei were stained with the nuclear stain DAPI (blue). Cells with >5 RAD51 foci were counted as RAD51-positive (RAD51+) cells from three fields of on each slide and the percentage of RAD51+ cells are plotted on the right. All experiments were repeated at least thrice and representative images are shown. **d** XTT growth assay on parental and PrexR cells after 48 h treatment with Prex (10 nM) with or without Olap (20 µM). *P < 0.05; **P < 0.01; ***P < 0.001; NS not significant.

Efficient DNA damage repair response is critical for survival in CHK1i-resistant cells during the delayed G2 phase. It has been shown that toxicity to CHK1i (Prex) monotherapy occurs via loss of CHK1 control over CDK1 activity leading to unscheduled mitotic entry of cells with unrepaired DNA damage [8, 13]. Consistently, our results showed increased levels of γH2AX-S139, a DNA DSB marker in both parental cell lines with 20 nM Prex treatment over 6–24 h, but no such increase was observed in PrexR cells treated with CHK1i (Fig. 4a). These trends were confirmed by immunofluorescence staining for γH2AX (Fig. 4b). This lack of DSBs is suggestive of DNA damages being repaired before they progress into DSBs in PrexR cells, possibly aided by the prolonged G2 delay and SSB repair prior to mitotic entry as shown in normal untreated cells. We therefore hypothesized that since CHK1i’s inhibition of active CHK1 remained unaffected in PrexR (Fig. 1f), CHK1 still could drive DSB repair via an active HR.

To investigate this, we evaluated RAD51, an HR marker, by immunoblotting and immunofluorescence microscopy. We previously reported that induction of nuclear RAD51 foci by PARPi olaparib was significantly attenuated by CHK1i Prex in ovarian cancer cells, thus sensitizing BRCAwt HGSOC to PARPi via causing an HR-deficient phenotype [7]. In immunoblots, no significant differences in total RAD51 levels were observed at baseline between parental and PrexR cells (Fig. 4a). While CHK1i reduced RAD51 levels in parental cells, it did not substantially affect total RAD51 levels in PrexR cells (Fig. 4a). Instead, we observed similar increases in RAD51 foci formation by sublethal concentrations of olaparib in both parental and PrexR cells. Moreover, CHK1i reversed this in both parental and PrexR cells similarly (Fig. 4c) suggesting that CHK1’s control over RAD51-mediated HR was preserved in PrexR cells. Growth assays were used to further confirm that increased toxicity in both parental and PrexR cells with or without Prex was independent of the concentrations of olaparib (Fig. 4d).

**Fig. 5:**
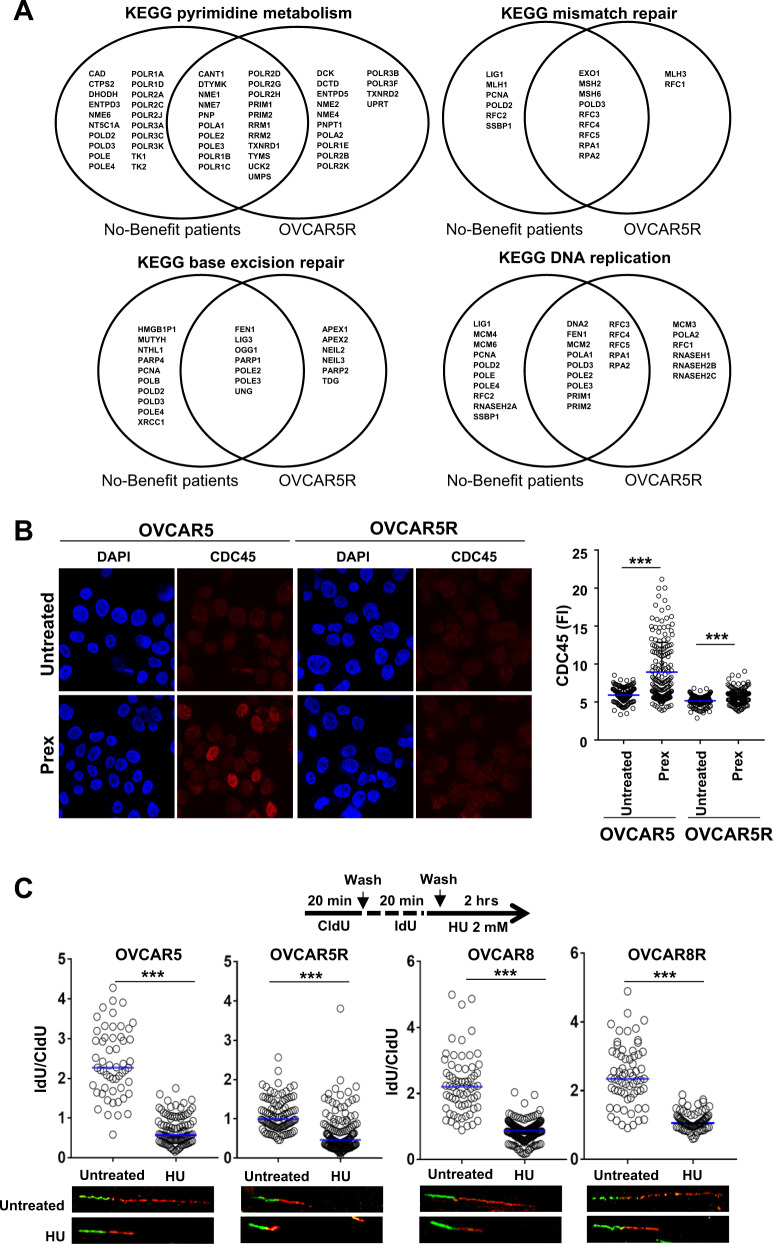
Roles of the replication fork and DNA repair pathways in CHK1i resistance. **a** Venn diagram plots of genes that contribute to the enrichment of gene sets as represented in Table 1. Common genes between the no-benefit patient group (*n* = 7) and the Prex-resistant OVCAR5R (*n* = 3) appear at the intersection between the two groups. **b** Immunofluorescence staining for DNA replisome protein CDC45 after pre-extraction was performed in OVCAR5 and OVCAR5R cells cultured overnight with or without Prex (20 nM). Fluorescence intensity (FI) for CDC45 was quantified for at least 200 cells by using ImageJ and plotted on the right. **c** DNA fiber assays were done on parental and PrexR cells. Cells were stained with CldU and IdU, followed by treatment with 2 mM HU for 2 h. Resection of nascent DNA strands induced by HU treatment (IdU labelled) was estimated as a ratio of IdU labelled tracts to CldU for at least 100 strands and plotted as median at 95% CI. Images of representative strands are shown below each plot. All experiments were repeated at least in triplicate and representative data is shown. All experiments were performed at least thrice. Data are shown as mean ± SD. ***P < 0.001; NS not significant.

To identify other DNA repair pathways that may contribute to DNA repair in a CHK1i-resistant setting, we performed gene set enrichment analysis (GSEA) of RNA-seq data from total RNA of OVCAR5 and OVCAR5R cells. These data were compared with an RNA-seq dataset from on-treatment tumor core biopsies of BRCAwt HGSOC patients (*n* = 12). Of twelve biopsied patients, seven demonstrated resistance to CHK1i therapy, defined as progressive disease (PD) or stable disease (SD) lasting less than 6 months (hereafter referred to as no-benefit patient group) [8]. Essential single-stranded DNA (ssDNA) repair pathways such as base excision repair (BER), mismatch repair (MMR), and those of DNA replication and pyrimidine metabolism pathways were enriched in both OVCAR5R vs. OVCAR5 cell lines and clinical samples in the no-benefit group vs. benefit group (Table 1). Further, overlap analysis of gene profiles showed 41–80% overlap for the different pathways between OVCAR5R and patient datasets (Fig. 5a). Together, our findings suggest that activities of ssDNA damage repair pathways remain intact in PrexR cells and HGSOC patient samples despite CHK1i treatment, which in concert with increased G2 delay driven by lower CDK1 activity, may contribute to resistance to CHK1i by helping repair DNA damage before they progress to DSBs.

**Table 1:** GSEA on gene sets that showed enrichment in patients that showed no clinical benefit (Progressive disease [PD] + stable disease [SD] <6 months) vs. benefit (PD + SD ≥6 months) compared with gene sets enriched in Prex-resistant OVCAR5R cell line vs. parental OVCAR5.

No benefit vs. benefit	OVCAR5R vs. OVCAR5
RNA polymerase	Neuroactive ligand receptor interaction
**Pyrimidine metabolism**	Sphingolipid metabolism
Spliceosome	Calcium signaling pathway
**DNA replication**	**Mismatch repair**
**Base excision repair**	Glycosylphosphatidylinositol gpi anchor biosynthesis
Lysine degradation	Purine metabolism
**Mismatch repair**	Glycosaminoglycan biosynthesis heparan sulfate
Nucleotide excision repair	Dilated cardiomyopathy
One carbon pool by folate	Basal transcription actors
Valine leucine and isoleucine degradation	**DNA replication**
Propanoate metabolism	Hedgehog signaling pathway
RNA degradation	**Pyrimidine metabolism**
Butanoate metabolism	**Base excision repair**
Thyroid cancer	Gap junction
Systemic lupus erythematosus	Basal cell carcinoma
Proteasome	Oocyte meiosis
Cysteine and methionine metabolism	Pathogenic *Escherichia coli* infection
N glycan biosynthesis	Hypertrophic cardiomyopathy hcm
Vasopressin regulated water reabsorption	Tight junction
Protein export	P53 signaling pathway

### Replication fork protection is not associated with Prex resistance

Prex induces replication stress and mitotic catastrophe thus causing cell death in HeLa cells [13]. To assess replication stress in PrexR cells, we first measured chromatin-bound CDC45 by immunofluorescent microscopy [21]. The initiation and elongation factor CDC45 is an essential rate-limiting component of replication progression complexes that assemble at active replication origins [22]. CDC45 accumulates at unscheduled fired origins (active origins) following CHK1i treatment [13]. We observed increased CDC45 staining in OVCAR5 parental cells treated with CHK1i (Fig. 5b, left) (*P* < 0.001) indicating augmented unscheduled origin firings. In contrast, CDC45 positive cells did not increase in OVCAR5R despite CHK1i treatment, suggesting no enhanced replication stress in PrexR cells (Fig. 5b, right) which is in line with no significant DNA damage in PrexR cells (Fig. 4b).

A component of stalled replication forks is the protection of ssDNA by replication protein A (RPA) to prevent nucleolytic degradation [13]. King et al. reported excessive unscheduled replication forks by Prex consequently depletes the cellular pool of RPA [13]). GSEA (Fig. 5a) of the KEGG MMR gene set, showed the enrichment of RPA transcripts as a common event between both PrexR and the no-benefit patient group. We therefore investigated whether increased replication fork protection is associated with the lack of replication stress and DNA damage in PrexR cells. We found higher basal levels of RPA70 protein (RPA 70 kDa DNA-binding subunit; RPA1) in PrexR cells compared with parental cells by immunofluorescent analysis (*P* < 0.001, Supplementary Fig. 4a, b) but no increase of RPA70 in PrexR cells after CHK1i treatment.

Next, using DNA fiber assays, we examined whether CHK1i resistance involved enhanced protection of stalled replication forks and whether this is partly mediated by endonuclease activity that is essential for HR repair of DSBs and stalled replication forks [23]. The replication fork poison HU induces DSBs and stalled replication forks followed by rapid recruitment of the endonuclease MRE11 to nuclear foci [24]. HU treatment of both parental and PrexR cells showed similarly shortened IdU strands (Fig. 5c) suggesting similar levels of stalled replication with or without Prex. Growth assays showed similar levels of toxicity as well when compared with untreated condition (Supplementary Fig. 5a), indicating no increased replication fork protection or DSB repair in PrexR cells. To further examine whether the absence of stalled replication forks (Fig. 5b) is associated with enhanced MRE11 activity and DSB repair in PrexR, we directly examined the effect of MRE11 inhibition in PrexR cells. Treatment with Mirin, an MRE11 inhibitor, showed similar toxicity profiles for both parental and PrexR cells (Supplementary Fig. 5b) with or without Prex. Together, these data suggest that CHK1i resistance is unlikely to be associated with the protection of stalled replication forks or enhanced DSB repair but more likely associated with upstream ssDNA damage repair pathways such as BER or MMR.

### Overcoming CHK1i resistance in HGSOC

**Fig. 6:**
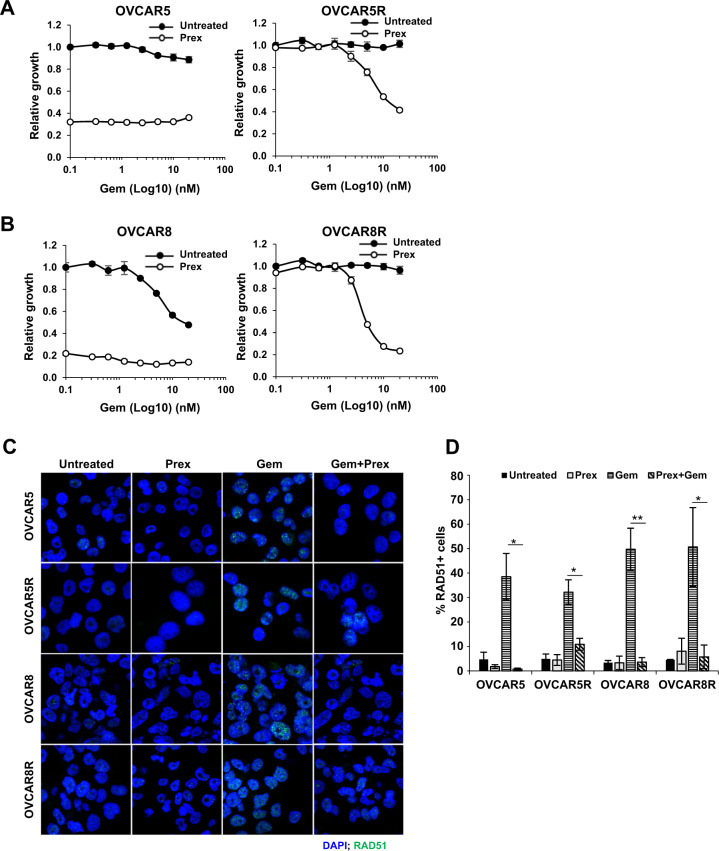
Effect of DNA damaging agents on CHK1i resistance. **a**, **b** XTT growth assays of parental and PrexR cells of OVCAR5 (**a**) or OVCAR8 (**b**). Cells were treated with gemcitabine (Gem) (0–20 nM) with or without Prex (10 nM) for 48 h. **c** Immunofluorescence staining of RAD51 was conducted on parental and PrexR cells treated overnight with either Prex (10 nM) or Gem (10 nM) or in combination. **d** Cells with >5 RAD51 foci were counted as RAD51+ cells and average count from three independent experiments are plotted. *P < 0.05; **P < 0.01; ***P < 0.001; NS not significant.

**Fig. 7:**
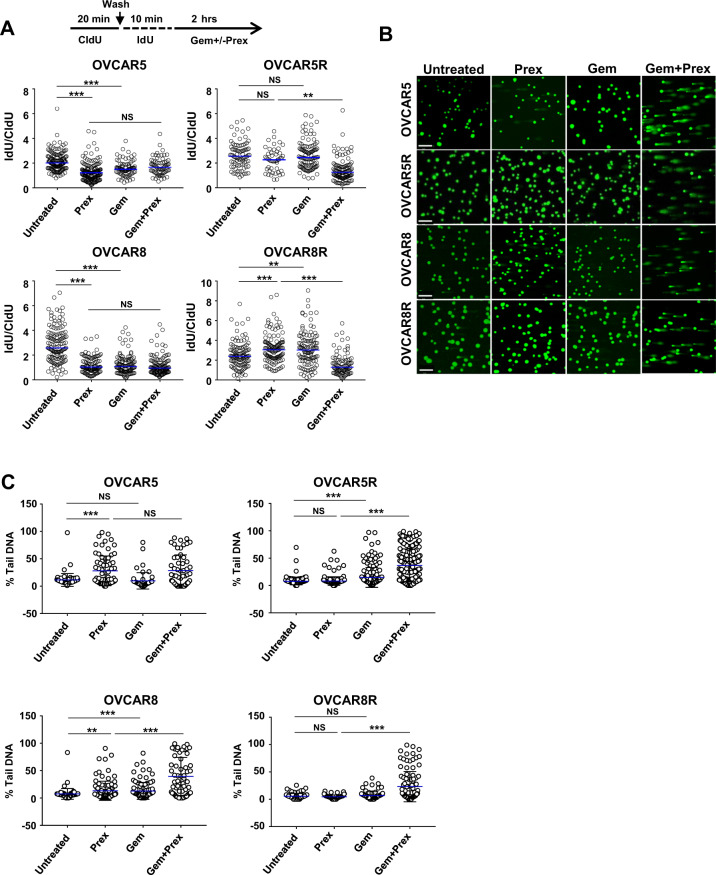
Replication fork stalling and DNA fragmentation upon combination treatment. **a** DNA fiber assay to measure replication fork stalling was performed on cells treated with CldU (20 min), IdU (10 min) followed by the addition of Gem (50 nM) or Prex (50 nM) or both for a further 2 h in the presence of IdU. The ratio of nascent IdU labeled strands to CldU labeled strands are plotted as a measure of stalled replication forks. **b**, **c** Alkaline comet assay to quantify DNA fragmentations in cells treated with Prex or Gem or in combination. At least 100 cells events were quantified using CometScore^©^ software. Percentage of tail DNA is plotted. Each experiment was repeated at least twice. *P < 0.05; **P < 0.01; ***P < 0.001; NS not significant.

Based on our data, we hypothesized that if CHK1’s control over HR remained intact in PrexR cells, CHK1i could induce RAD51-mediated HR inhibition (Fig. 4c) thus sensitize PrexR cells to DNA damaging agents causing DSBs. To test this hypothesis, we treated parental and PrexR cells with 10 nM Prex and sublethal concentrations (1–20 nM) of gemcitabine (Fig. 6a, b). Growth assays over 48 h showed expected Prex sensitivity of both parental lines with only OVCAR8 showed sensitivity to gemcitabine (Fig. 6b, left). Both PrexR cells demonstrated no loss of viability to each Prex and gemcitabine monotherapy (Fig. 6a, b, right untreated). In combination, Prex treatment induced a concentration-dependent sensitization to gemcitabine (Fig. 6a, b, right Prex) in PrexR cells. Immunofluorescent microscopy (Fig. 6c, d) further confirmed that Prex did indeed inhibit RAD51 foci formation by gemcitabine in both PrexR and parental cells uniformly (Fig. 4c). Further, DNA fiber assays showed an increased frequency of stalled replication forks with CHK1i and gemcitabine combination in both parental and PrexR cells as indicated by a lower IdU/CldU ratio compared with untreated control (Fig. 7a), while only parental cells showed increased replication fork stalls with CHK1i or gemcitabine monotherapy, further supporting our earlier observations.

Lastly, we performed an alkaline comet assay to evaluate whether the increased replication fork stalls by combination treatment could be translated into the enhanced DNA damage in PrexR cells. We found CHK1i and gemcitabine combination augmented DNA fragmentations (*P* < 0.01) in both parental and PrexR cells, (Fig. 7b, c) while no increase in comets was observed with CHK1i alone in PrexR cells. Also, as anticipated, CHK1i or gemcitabine monotherapy induced a significant increase in comets in parental cells. Overall, our data suggest a functional separation of CHK1 activities that has distinct roles in Prex resistance and sensitization to DNA damaging agents in combination therapy.

## Discussion

Cell cycle checkpoints, e.g., ATR and CHK1 are active therapeutic targets in numerous cancers including HGSOC either as a monotherapy or as a sensitizer of DNA damaging drugs and radiation therapy [25–27]. In order to effectively advance this class of drugs to novel clinical trials for HGSOC, investigating critical steps involved in CHK1i resistance is necessary. In this study, we identified that lower CDK1/CyclinB1 activity in BRCAwt HGSOC cells confers resistance to CHK1i by keeping cells from entering early mitosis with under-replicated DNA, thus preventing consequent mitotic catastrophe. CHK1i-resistant cells circumvent CHK1i-induced mitotic catastrophe and cell death by instituting a CHK1-independent G2 delay and a sustained DNA damage response. Notably, CHK1’s control over HR remained intact despite the development of resistance, which makes its role in HR distinct from regulating CDK1 activity, thus opening new vistas in understanding and overcoming Prex resistance.

CHK1i forces cancer cells into premature mitotic entry without optimal DNA repair, leading to replication catastrophe and cell death [26]. Thus, resistance mechanisms against other DNA damaging agents have been variably associated with arrests at G2 phase or with overcompensated DNA repair [28–30]. But, while Ruiz et al. showed CDC25A levels positively correlated with sensitivity to an ATR inhibitor based on the genome-wide CRISPR screen using murine embryonic stem cells [31], we did not find low levels of CDC25A in CHK1i-resistant HGSOC cells or patient samples (data not shown). Instead, a large subpopulation of G2 cells was CyclinB1-negative along with the low levels of mitotic marker pHH3-S10. The differential effects of ATR, CHK1, and WEE1 on DNA replication may present different mechanisms of resistance to ATR, CHK1, and WEE1 inhibitors although all involved the delayed G2 phase [32], warranting further investigation. Also, given that *BRCA* mutation status is associated with PARPi resistance/sensitivity in HGSOC [6, 7], it is imperative to study potential mechanisms of resistance to CHK1i separately for *BRCA* mutant HGSOC models.

The known roles of CDK1/CyclinB1 in regulating G2/M transition and resistance to DNA damaging agents [20, 28] led us to assess their modulations in CHK1i-resistant BRCAwt HGSOC. We found overall low levels of CyclinB1 in PrexR cells, a large CyclinB1-negative G2 population that was mitotically less active relative to parental cells. Further, siRNA-based silencing of CCNB1 partially recapitulated CHK1i-resistant phenotype in parental cells, similar to the reports in prostate cancer [33] or breast cancer models [34]. Interestingly, inhibition of extranuclear export of CyclinB1 with leptomycin B or overexpressing CyclinB1 itself did not reverse the resistance to Prex treatment suggesting multiple factors play the roles in developing resistance to CHK1i e.g., the available CDK1 for an active CDK1/CyclinB1 complex formation, the requirement of CyclinB1 nuclear localization and corresponding cell cycle phase for CyclinB1’s functional execution [19]. Consistently, CDK1 activity was much lower in PrexR cells and a specific inhibitor of CDK1 recapitulated G2 delay in parental cells like what we observed in PrexR cells.

While our data indicate that reduced CDK1 activity is a key contributory factor of CHK1i resistance in BRCAwt HGSOC, this G2 delay should be orchestrated with active DNA repair response for cell survival. It also suggests that normal CyclinB1 levels are essential for DNA damage to occur with CHK1i. In line with this, our transcriptome analysis and GSEA data of OVCAR5R cells and PrexR patient samples showed similar enrichment of genes related to essential DNA repair pathways such as BER, MMR, and other ssDNA damage repair pathways as well as increased RPA transcripts. Enhanced stabilization of ssDNA at stalled replication forks by RPA is vital for efficient DNA repair [35] and has been implicated in resistance to platinum drugs in ovarian cancer [36]. Our subsequent experiments indicated that RPA-related ssDNA protection or increased endonuclease activity, crucial for efficient DNA resection and DSB repair at stalled replication forks, was unsustainable when further DNA damage occurred with HU in PrexR cells. We therefore concluded that fork stability in PrexR cells is not a major cause of CHK1i resistance.

Another new finding of the present study is that CHK1i treatment reduced RAD51 foci formation by PARPi or gemcitabine not only in parental cells but also in PrexR cells. It is notable that CHK1i treatment still mitigates RAD51-mediated HR in PrexR cells, thus sensitizing PrexR cells to DNA damaging agents that require HR for repair. Our finding on the combination treatment approach in PrexR models is consistent with the previous report in pediatric cancer preclinical models [37] and further provides a rationale as to how the combination therapy can circumvent the resistance to Prex monotherapy.

In summary, our study demonstrates novel mechanistic insights of functionally distinct CHK1 activities in BRCAwt HGSOC; first, its role in G2/M checkpoint and secondly, in regulating HR. Our data therefore highlight a combination treatment strategy to overcome CHK1i resistance in BRCAwt HGSOC, warranting further investigation of these endpoints in relevant in vivo settings.

## Materials and methods

### Cell growth assays by XTT

This was done as detailed earlier [38]. Plates were read on a BioTek SynergyHT™ plate reader (BioTek Instruments, VT) and analyzed on Gen5™ software. Absorbance measured at 490 nm was plotted as absolute values (corrected for background) or relative to untreated control.

### Immunoblotting and subcellular fractionation

Immunoblotting was performed as described [7]. Blots were visualized and documented on an Odyssey™ Fc gel documentation system (LI-COR biosystems, NE).

### Cell cycle analysis

DNA content measurement was performed [39] and analyzed on a BD FACScanto™II (BD Biosciences, CA) and FlowJo software (FlowJo LLC, MD). For flow cytometric analysis of CyclinB1 and mitotic marker phospho-HistoneH3 (pHH3-S10), harvested cells were first permeabilized with 0.1% Triton X100 in phosphate-buffered saline (PBS), stained with a rabbit α-CyclinB1 and goat α-rabbit-AF488 secondary antibody and mouse α-pHH3-S10-AF647 antibody prior to fixation and PI staining.

### Quantitative PCR (qPCR) analysis

Total RNA was isolated from cells using RNeasy™ Micro kit (Qiagen, MD). Single-stranded cDNA was generated using the Superscript™ First-strand synthesis system (Thermo Fisher Scientific, MA). Primers specific for CyclinB1 mRNA (*CCNB1*) Forward 5′-CAGATGTTTCCATTGGGCTT-3′ and reverse 5′-TACCTATGCTGGTGCCAGTG-3′ and for endogenous control *GAPDH* were obtained from Integrated DNA Technologies, IA. qPCR was performed on an ABI ViiA7 Real-Time PCR system (Applied Biosystems, CA) and analyzed with QuantStudio™ Real-Time PCR software.

### Small interfering RNA (siRNA) transfection

A pool of four specific siRNAs (OnTargetPlus™ smartpool) against CyclinB1 (Dharmacon Inc, CO) was used for transfection with Lipofectamine 2000™ reagent (Thermo Fisher Scientific) as per manufacturer’s protocol. Cells were transfected for at least 48 h before use for growth assays or western blot.

### Co-immunoprecipitation and CDK1 kinase activity assays

Lysates prepared from 1 × 10^7^ cells in lysis buffer (50 mM Tris-HCl, pH8.0, 150 mM NaCl, 1% NP-40 containing complete™ and PhosSTOP™[Roche]) were incubated with rabbit α-CDC2 (α-CDK1) (#28439, Cell Signaling Technology, MA) (1:50) overnight at 4 °C and captured using protein G-agarose (Thermo Fisher Scientific) as per manufacturer’s protocol. Immunoprecipitates were washed and resuspended in 25 µl of kinase buffer (40 mM HEPES, pH 7.5, 10 mM MgCl_2_, 1 mM EGTA, 0.01% Brij35), containing Histone H1 (5 µg) protein (Sigma-Aldrich) and 50 µM of ATP. After incubation at 31 °C for 1 h, the reaction was stopped with 25 µl of 1× Laemmli buffer, boiled for 5 min, and used for immunoblotting.

### Immunofluorescence microscopy

Cells were prepared as detailed earlier [7]. For RAD51 immunofluorescence, coverslips were further stained with DAPI, mounted using Eukitt medium as reported [40] before acquisition and analysis with a Zeiss 780 laser confocal microscope and Fiji (is ImageJ)™ (National Institutes of Health, MD). Cells with >5 RAD51 foci were counted as positive and an average of three experiments was plotted with error bars showing the sample error of the mean (SEM). For CDC45 immunofluorescence, cells on coverslips were incubated with 0.1% Triton-X 100/PBS for 1 min on ice, and then fixed with 4% paraformaldehyde. Signal intensity of CDC45 in each cell was quantified with ImageJ.

### RNA-seq

RNA-seq was performed using tumor core biopsy samples from 12 HGSOC patients on the CHK1i Prex clinical trial (ClinicalTrials.gov, NCT02203513) as detailed before [8]. Datasets were deposited in NCBI’s Gene Expression Omnibus database under accession numbers GSE149723 and GSE149724. More details are provided in Supplementary Methods.

### DNA fiber assay

Cells were plated at 40–50% density in six-well plates a day prior to treating sequentially with 5-Chloro-2′-deoxyuridine (CldU) and 5-Iodo-2′-deoxyuridine (IdU). To measure DNA resection, cells were first labeled with 60 µmol/L thymidine analogue CldU for 20 min, washed and labeled with 500 µmol/L IdU for 20 min, washed and then treated with 2 mM of HU for 2 h. To measure stalled DNA replication following DNA damage, cells were labeled with CldU for 20 min, washed and labeled with IdU for 10 min followed by gemcitabine or Prex (50 nM each) or both for 2 h. Labeled cells were harvested and DNA fiber spreads were prepared as described [41]. Slides were imaged using a Zeiss 780 laser microscope and at least 100 fibers per condition per experiment was measured and analyzed using ImageJ software. Data were plotted as median with 95% confidence interval (CI) (GraphPad Prism V7).

### Comet assay

Alkaline comet assays were performed as detailed [7]. Data were plotted as mean % tail DNA with error bars showing standard deviation (GraphPad Prism V7).

### Statistical analysis

Student’s *t* test was used to determine statistical significance for paired groups of data whereas an unpaired Mann–Whitney *t* test was performed for DNA fiber, comet assay, and RNA-seq data analyses (GraphPad Prism V7). *P* < 0.05 was significant. Minimum sample sizes for statistical significance were determined in consultation with biostatisticians.

## Supplementary information

Supplemental material
