# Supplementary material for: Resistance to the CHK1 inhibitor prexasertib involves functionally distinct CHK1 activities in BRCA wild-type ovarian cancer
Source: Oncogene. 2020 Jul 9;39(33):5520–35. doi: 10.1038/s41388-020-1383-4 (PMC7426265; doi:10.1038/s41388-020-1383-4)
Supplement: Supplementary file 1 — Supplemental material [file 41388_2020_1383_MOESM1_ESM.docx]

**Supplemental methods**

**Agents**

Inhibitors were purchased from the following companies: Prex (LY2606368, MedChem Express, NJ), HU (Sigma-Aldrich, MO), gemcitabine and Ro336 (both from SelleckChem, PA). Antibodies used for flow cytometric analysis include rabbit α-CyclinB1 (#ab32053, Abcam, MA), mouse α-pHH3-S10-AF647 (#650806) and goat secondary antibodies α-rabbit IgG-AF488 and α-mouse-IgG-AF647 (all from Life Technologies, CA). Antibodies used for western analysis were either from Cell Signaling Technology, MA (CHK1, CHK1-S296, CHK1-S345, CHK1-S317, GAPDH, CyclinB1, LaminA, Histone H1, CDC25A, CDC25C, pCDC25C-S216, γH2AX-S139, RAD51, pATR-S428, ATR), Abcam (pATM-12981, ATM, pCDC25A-S124), or from MyBiosource, MA (Histone H1-T154). Antibodies for immunofluorescent microscopy include α-H2AX (S139)-AF647 (#613408, Biolegend, CA), α-CDC45L (#ab126762, Abcam) and rabbit α-RAD51 (#ab133534, Abcam). For DNA fiber assay, DNA dyes 5-Iodo-2'-deoxyuridine (IdU) and 5-Chloro-2'-deoxyuridine (CldU) (Sigma-Aldrich) and their specific antibodies mouse α-IdU (#NBP2-44056, Novus) and rat α-CldU (#NB500-169, Novus Biologicals,CO) and corresponding secondary antibodies goat α-rat IgG-AF488 (#A11006, Thermo Fisher Scientific, MA) and goat α-mouse-AF488 (#A11005, Thermo Fisher Scientific) were used.

**Cell lines**

We developed CHK1i-resistant cell lines using BRCAwt HGSOC cells (OVCAR5 [p53 null] and OVCAR8 [p53 deletion mutant]) to actively reflect the platinum-resistant recurrent BRCAwt HGSOC patients treated on phase 2 clinical trial of CHK1i Prex (NCT02203513) [1]. Cell lines OVCAR5 and OVCAR8 were obtained from NCI-60 collection at the National Cancer Institute (National Institutes of Health, MD). All cell lines were cultured in RPMI1640 media with L-glutamine (Life Technologies) containing 10% FBS, 1000 U/ml penicillin/streptomycin, 1 mM sodium pyruvate and 5 µg/ml of insulin from bovine pancreas (Sigma-Aldrich). CHK1i Prex-resistant (PrexR) cell lines OVCAR5R and OVCAR8R were developed from their parental OVCAR5 and OVCAR8 [2] by culturing them in progressively increasing concentrations of Prex up to 3 µM over 3-4 months. PrexR cells were routinely maintained at 2 µM of Prex. Cells were cultured without Prex for at least 4 days until use.

**RNA-seq Analysis**

The clinical study protocol was reviewed and approved by the Institutional Review Board of the Center for Cancer Research, National Cancer Institute. Written informed consent was obtained from all patients. Separately, total RNA was also prepared from triplicate cultures of OVCAR5 and OVCAR5R and RNA-seq was performed as reported [1]. Datasets were quartile normalized and log-transformed prior to analysis. GSEA was performed using the GSEA tool on Genepattern™ [3] and the list of genes that contributed to enrichment within the selected datasets was analyzed using Microsoft excel.

**Growth curve and generation time**

Growth curve experiments were performed by seeding 1x10^4^ cells per well at Day 0 in 12-well plates in normal media (1 ml) and two wells were harvested and counted every day. Time of collection was noted, total cells per well estimated and growth curves were plotted after Day 10. Media was changed every 3-4 days. Generation time (GT) for each cell line was calculated by using cell line specific k = Ln(F/I)/T to calculate GT= Ln(2/1)/k where F and I are final and initial cell numbers in time T (dotted line) [4].

**Supplementary Figure Legends**

**Figure 1. Clonogenic assays were performed to show the persistence of CHK1i resistance in PrexR cells**

**A.** PrexR cells were cultured without Prex at indicated weeks (Wk). 2.5 x 10^3^ cells were re-seeded per well of a 6-well plate at the end of each week. Plates were further incubated for 7 days for the development of colonies and stained with 0.5% Crystal violet for 5 min before washing and imaging. **B.** Generation time assays were performed on both parental and PrexR cells in normal media over 10 days. This was performed once.

**Figure 2. Leptomycin B, a CyclinB1 efflux inhibitor and its effect on survival**

**A.** Parental and PrexR cells were plated on lysine coated coverslips and treated with leptomycin B (0.3 nM) overnight before imaging the cells for CyclinB1 as described in methods. **B.** Cell growth assays were performed using XTT in cells treated with a gradient of leptomycin B with or without Prex (5 nM) for 48 hrs. All experiments were repeated at least thrice. These experiments were repeated twice **C-E.** Parental and PrexR cells were cultured overnight to 50-70% confluency in 6 well dishes before transfecting with pCMX-CCNB1GFP expression vector using either Dharmafect Kb (OVCAR5) or Fugene (OVCAR8). After 24 hrs of transfection, cells were harvested, counted and used for either XTT assays for a further 48 hrs with or without Prex (0-100 nM for parental, and 0-500 nM for PrexR cells) (C) or for flow cytometric analysis following Prex (10 nM) treatment over 24 hrs (D), or plated on lysine coated coverslips and stained for γH2AX-AF647 and visualized as detailed in Methods (E). CCNB1-GFP expressing cells are fluorescent green in the image. This was performed twice.

**Figure 3. Immunoblots of CDK1 levels and CDK1 activity in parental and PrexR cells**

**A.** Western blot of histone H1 (HH1) and phosphorylated histone H1-T154 (pHH1-T154) were measured in parental and PrexR cells. Densitometric quantification of pHH1 is normalized with total HH1 (pHH1+HH1) and plotted on the right as a measure of CDK1 activity. **B.** Immunoblotting analysis of CDK1 and its inactive phosphorylated form (pCDK1-Y15) was performed on protein lysates prepared from cells that were treated with Prex (20 nM) for 6 hrs or 24 hrs. Densitometric measurements normalized to GAPDH is shown below as numbers. Experiments were performed at least thrice

**Figure 4. Analysis of RPA70 expression in parental and PrexR cells**

**A.** Cells were incubated with Prex (20 nM) overnight before staining with antibodies against RPA70 and counterstained with secondary antibodies conjugated to AF488 and DAPI. Images were acquired on a Zeiss 780 confocal microscope. **B.** RPA70 fluorescence intensity (FI) for each cell was quantified using ImageJ software and absolute FI plotted with Graphpad Prism. The columns show median with 95% CI. The experiment was repeated twice and representative images are shown. ***, *P* < 0.001; NS, not significant.

**Figure 5. Effect of replication toxin HU and Mirin, an MRE11 inhibitor on Prex sensitivity**

**A.** Cell growth assays were performed using XTT in cells treated with HU (0-0.5 mM) with or without Prex (5 nM) for 48 hrs. All experiments were repeated at least twice and representative images are shown. **B.** Cell growth rates were performed using XTT assays in parental and PrexR cells cultured with or without Prex (20 nM) and co-treated with a gradient (0-50 µM) of Mirin for 48 hrs. All experiments were repeated at least twice and representative figures are shown.

**Supplementary Figure 1.**


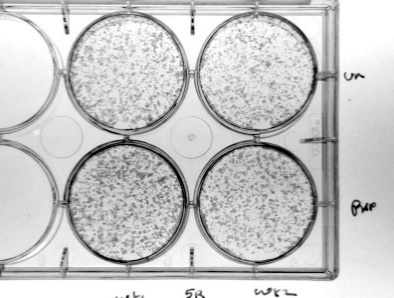


**OVCAR5R**

**Wk1 Wk2 Wk3 Wk4 Wk5 Wk6 Wk7**


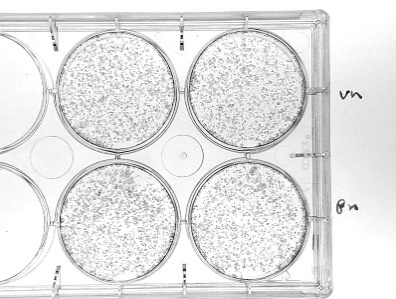


**OVCAR5**

**OVCAR8**


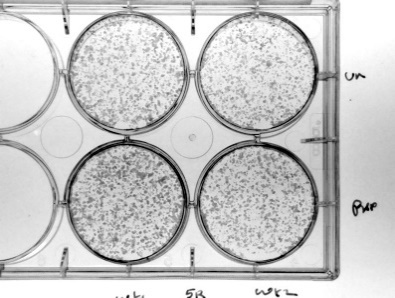

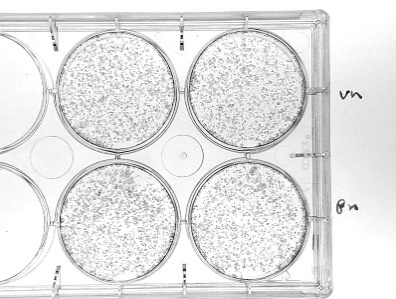


**DMSO**

**Prex (20 nM)**


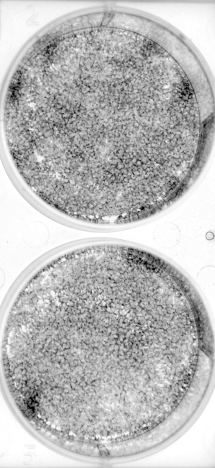

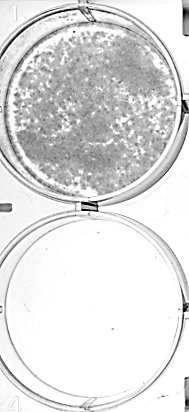

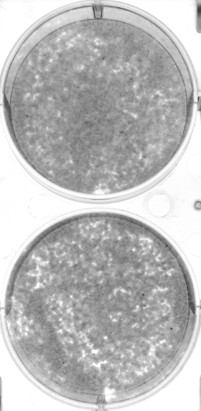

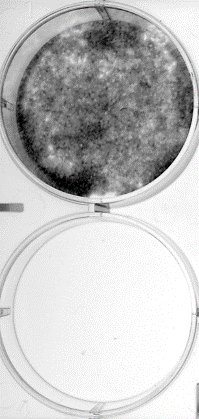

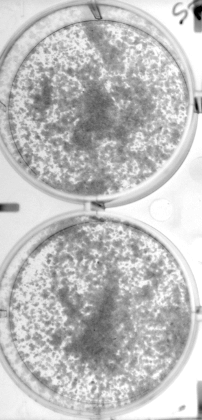

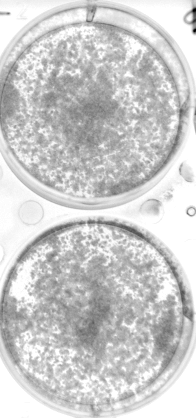

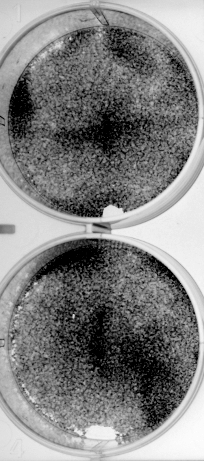

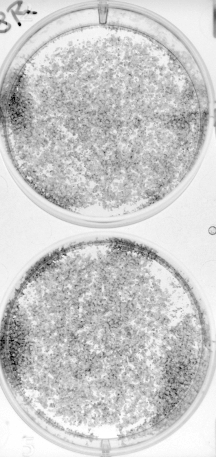

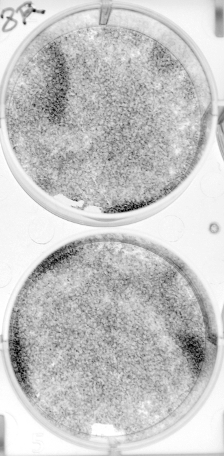

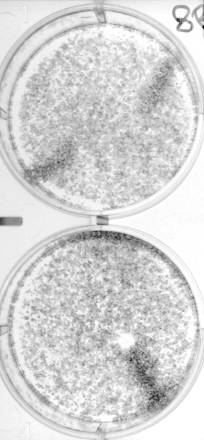

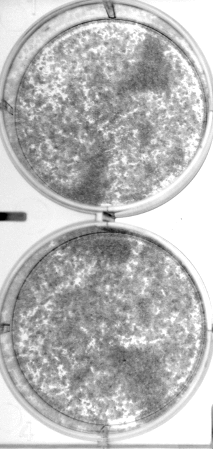

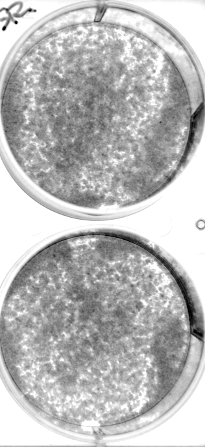


**DMSO**

**Prex (20 nM)**

**OVCAR8R**

**Wk1 Wk2 Wk3 Wk4 Wk5 Wk6 Wk7**

**A**


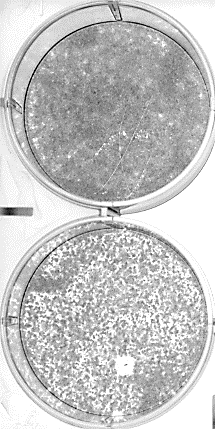


**OVCAR5R**

**OVCAR8R**

**DMSO**

**Prex (1 µM)**


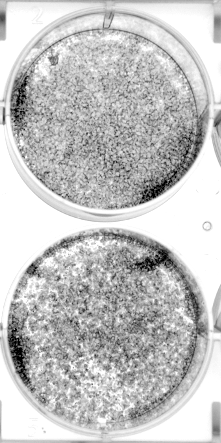


**Wk7**

**Wk7**

**B**

**Supplementary Figure 2.**

**A**


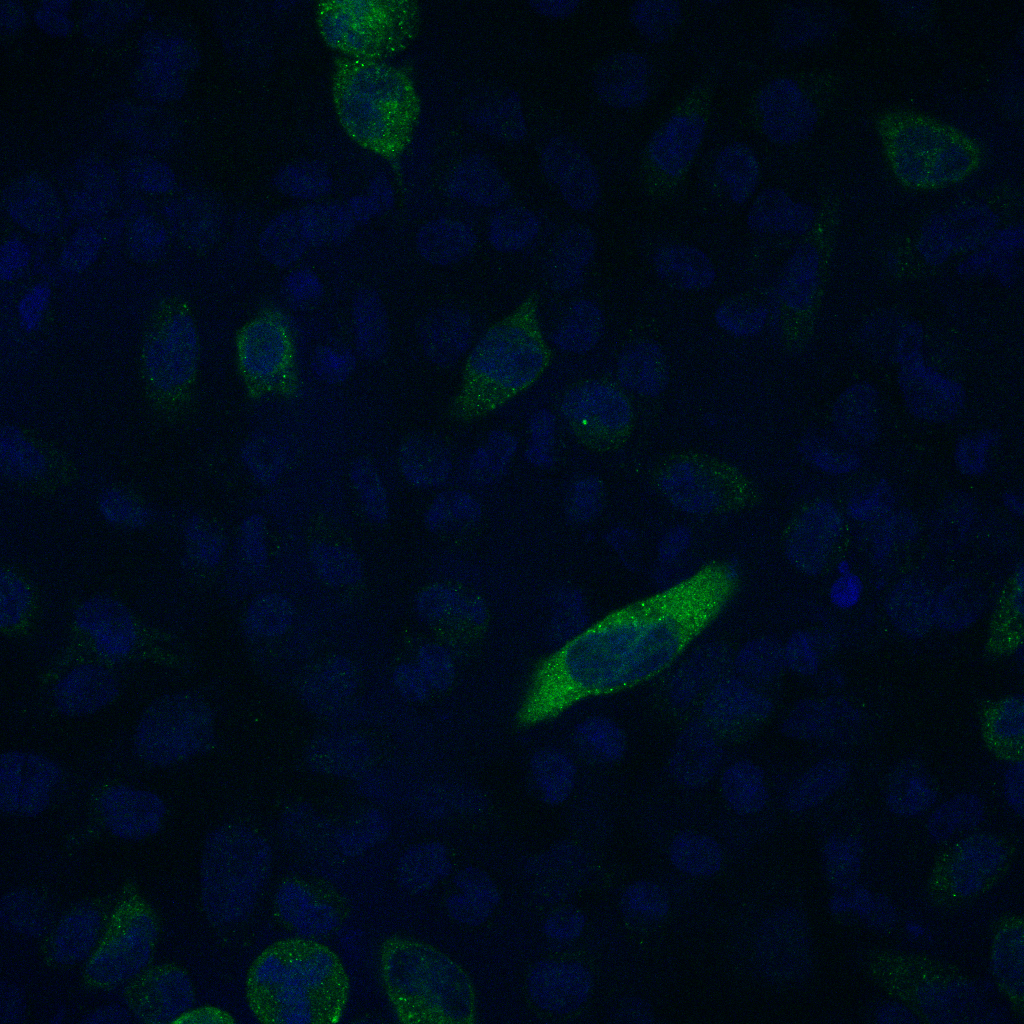

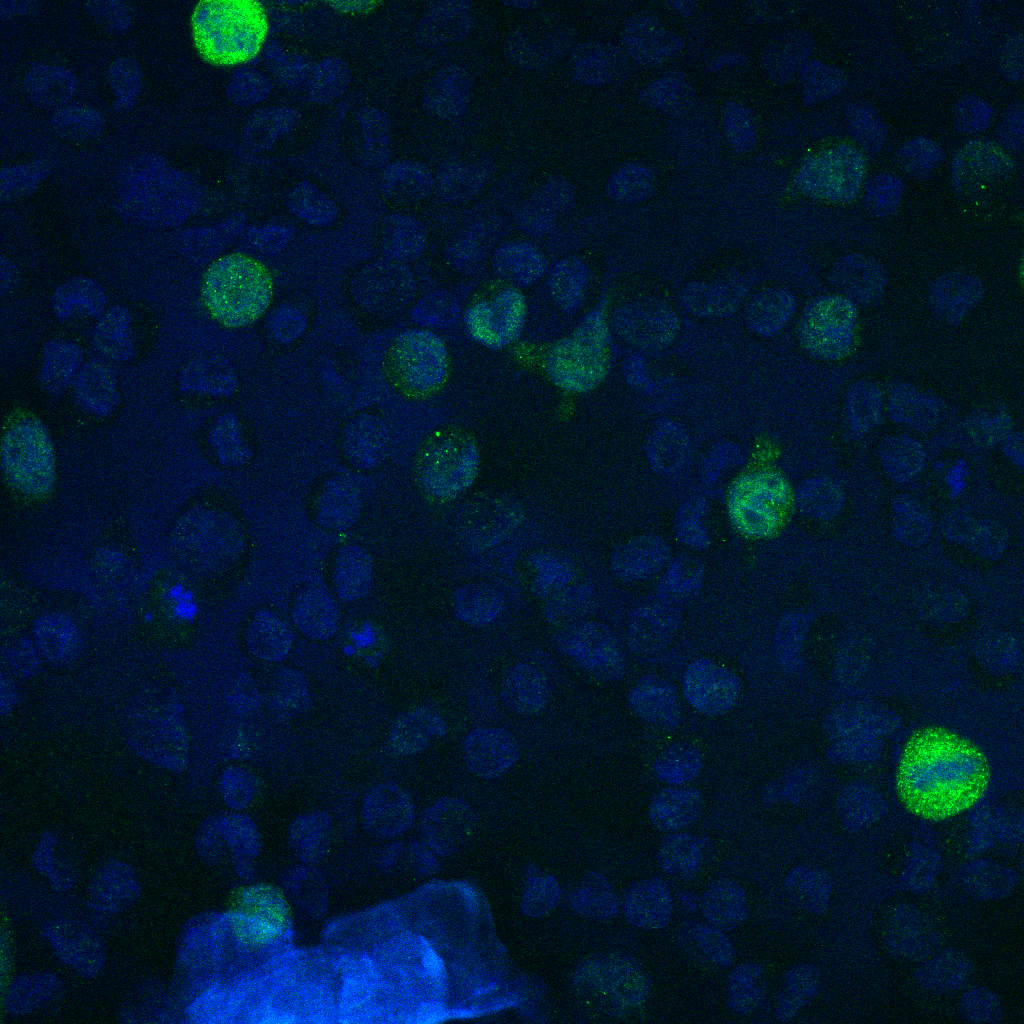

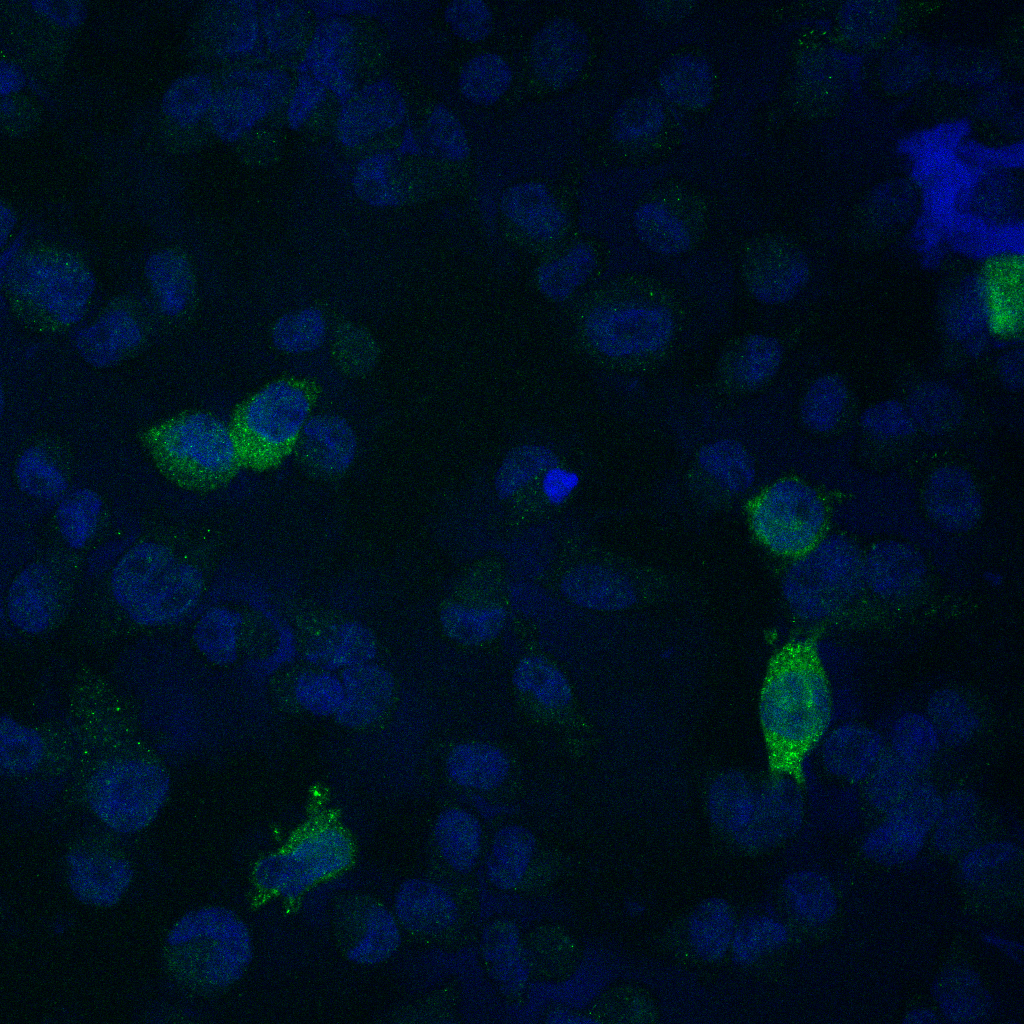


**OVCAR5**

**OVCAR8**

**Untreated**

**Leptomycin B**

**Untreated**

**Leptomycin B**


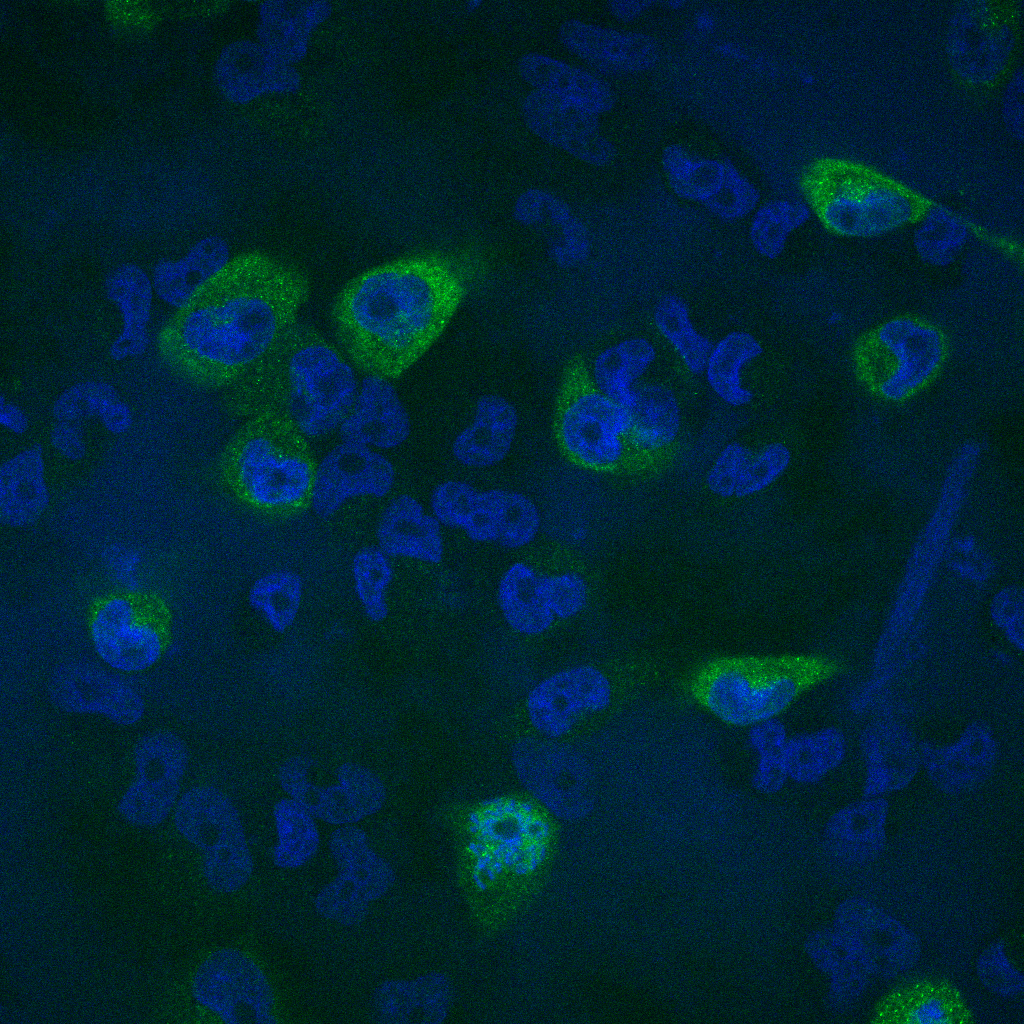

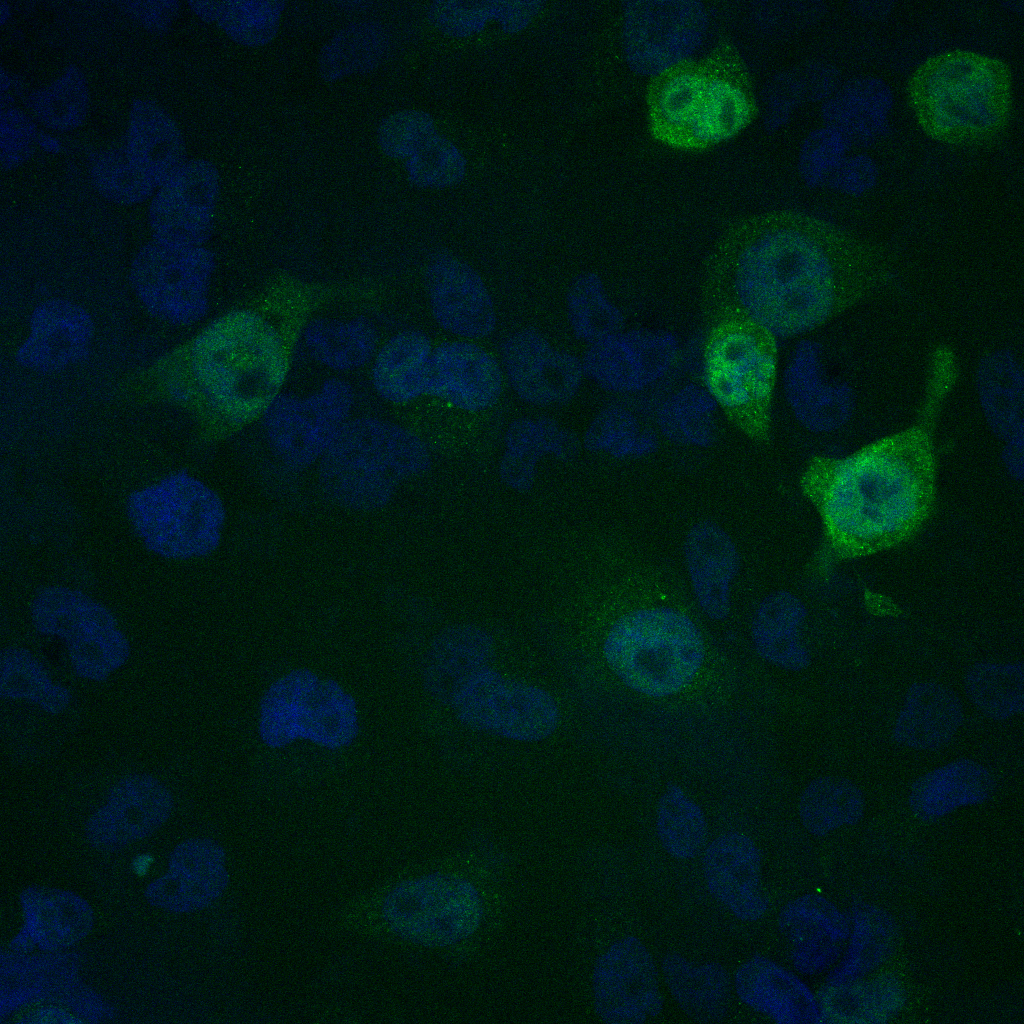

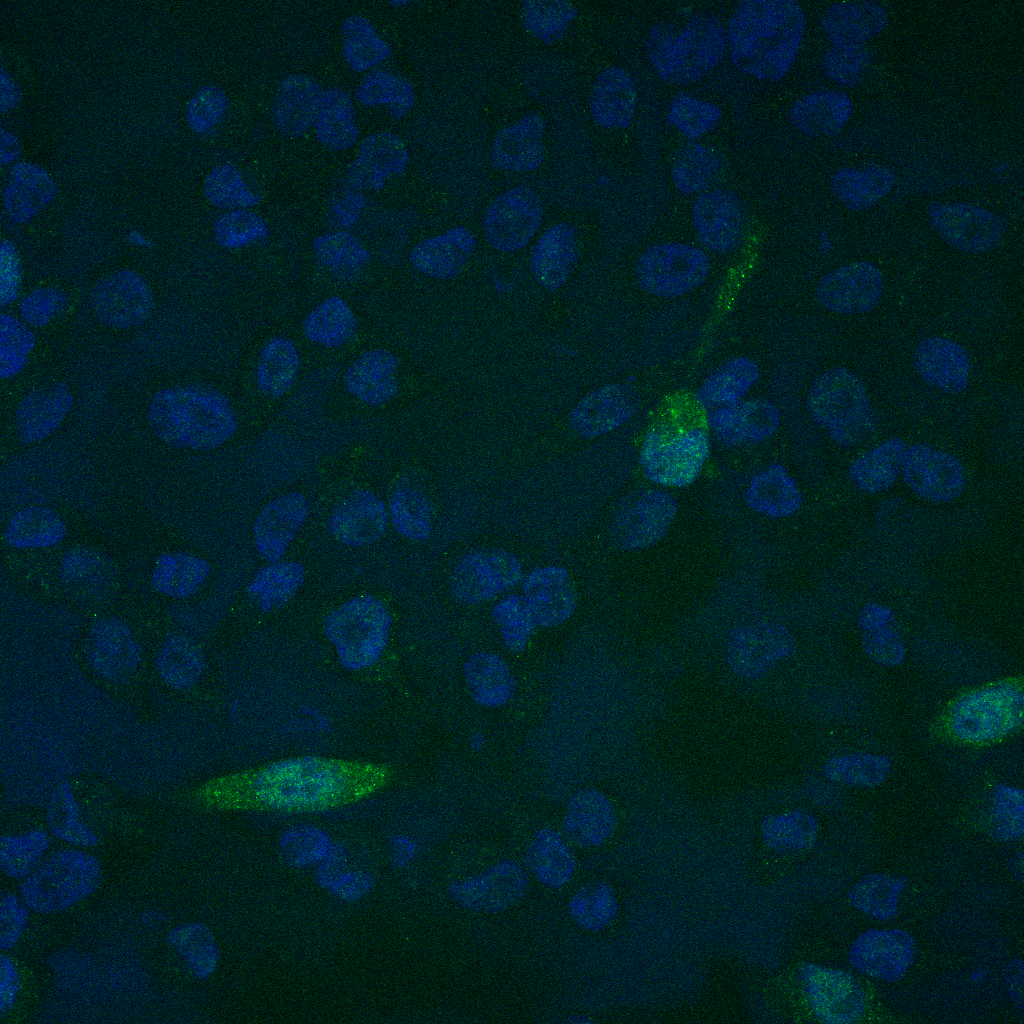

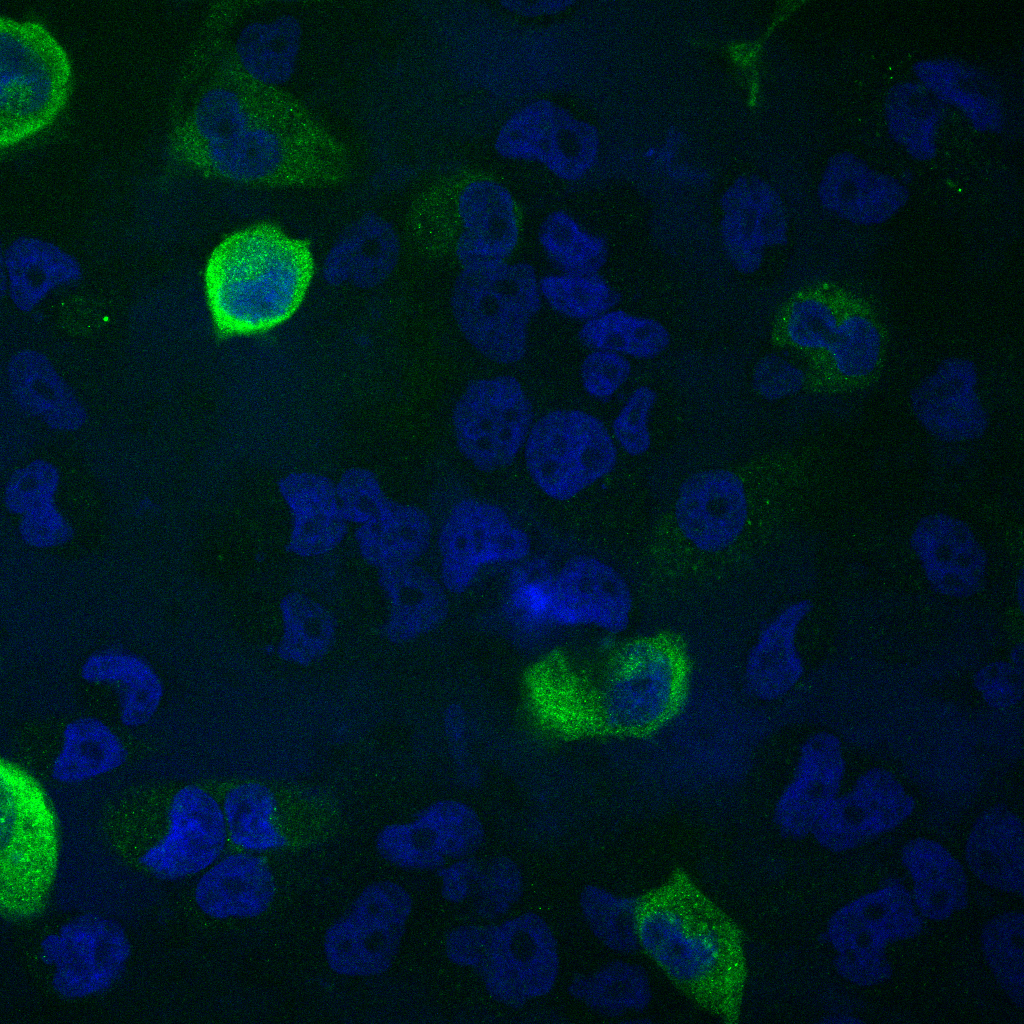


**OVCAR5R**

**OVCAR8R**


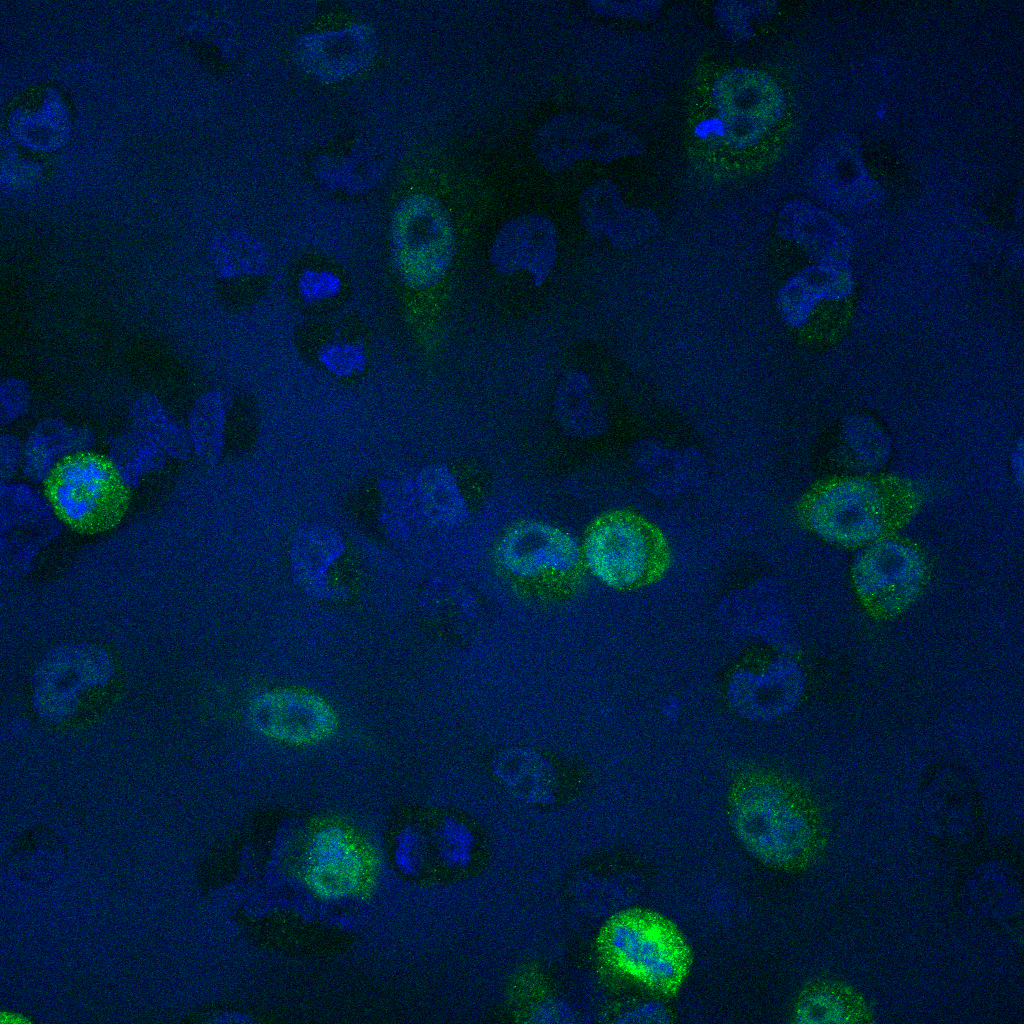


**DAPI; CyclinB1**

**B**

**C**

**Mock**


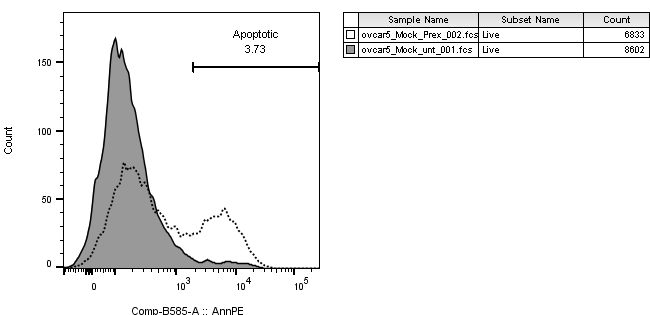


**pCCNB1**

Untreated

Prex

Apoptotic

3.7%

30 %


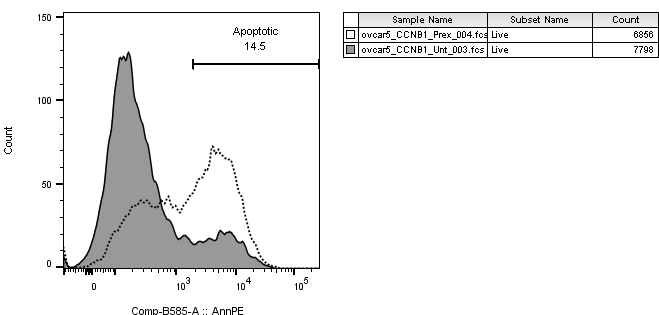


Apoptotic

15%

52 %

**OVCAR5**


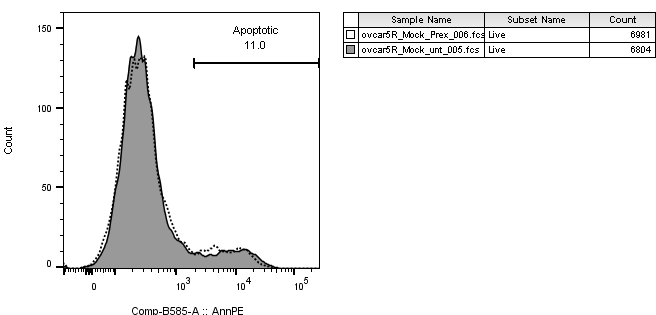


**OVCAR8**


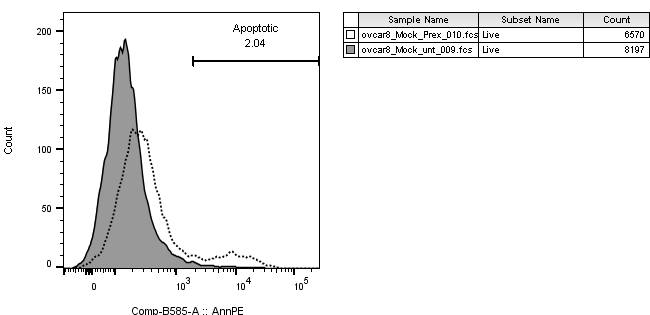


Apoptotic

2%

12 %


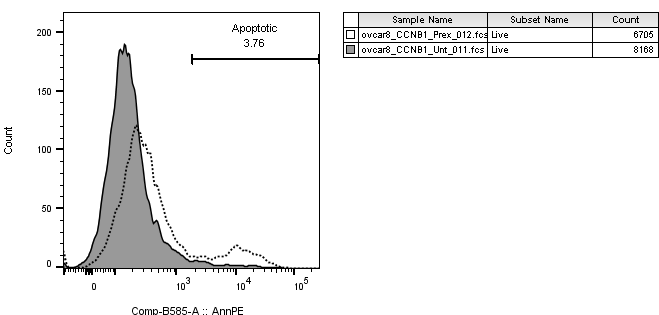


Apoptotic

3.8%

15 %


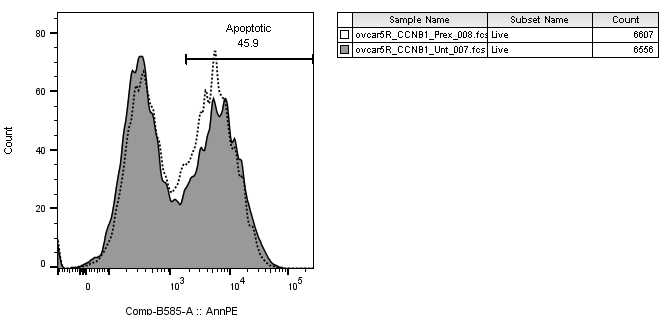

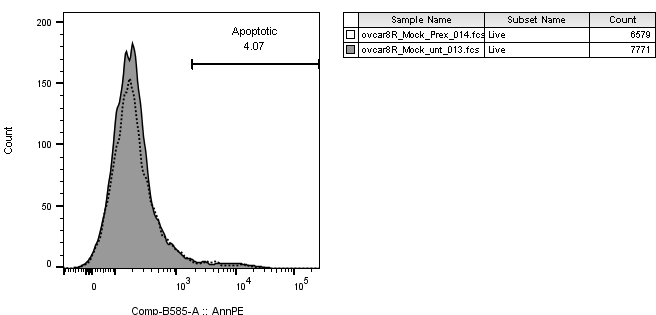

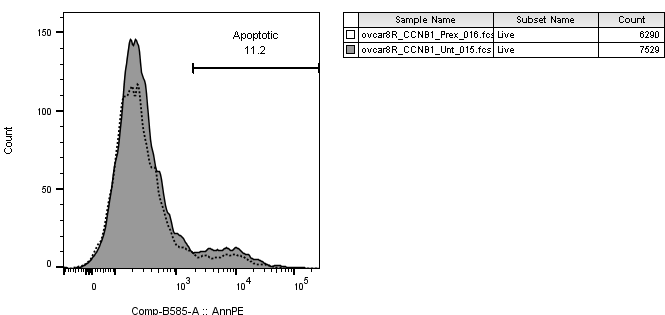


**Mock**

**pCCNB1**

Apoptotic

11%

11 %

Apoptotic

46%

49 %

Apoptotic

4.1%

4.2%

Apoptotic

11%

9 %

**OVCAR5R**

**OVCAR8R**

**AnnexinV -PE**

**D**

**Mock**

**Untreated**

**Prex**

**OVCAR8**

**OVCAR8R**


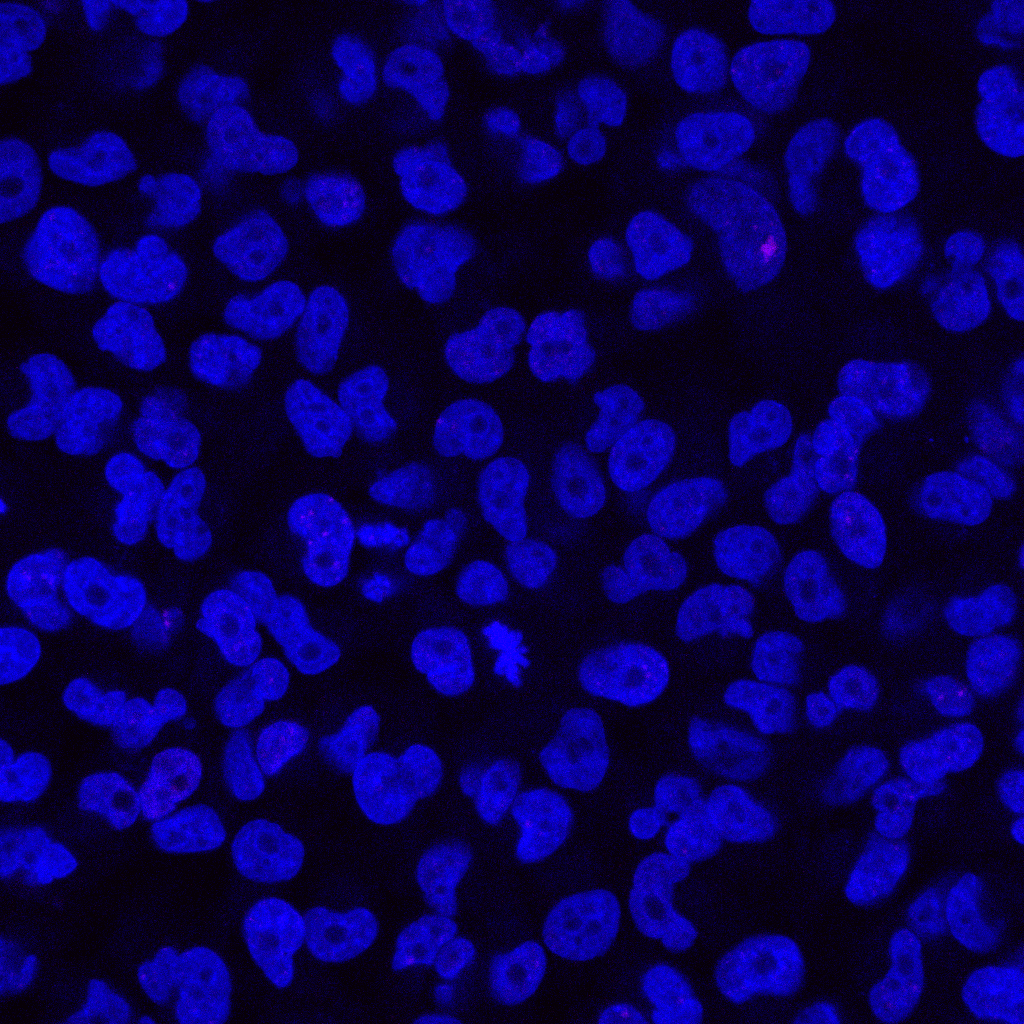

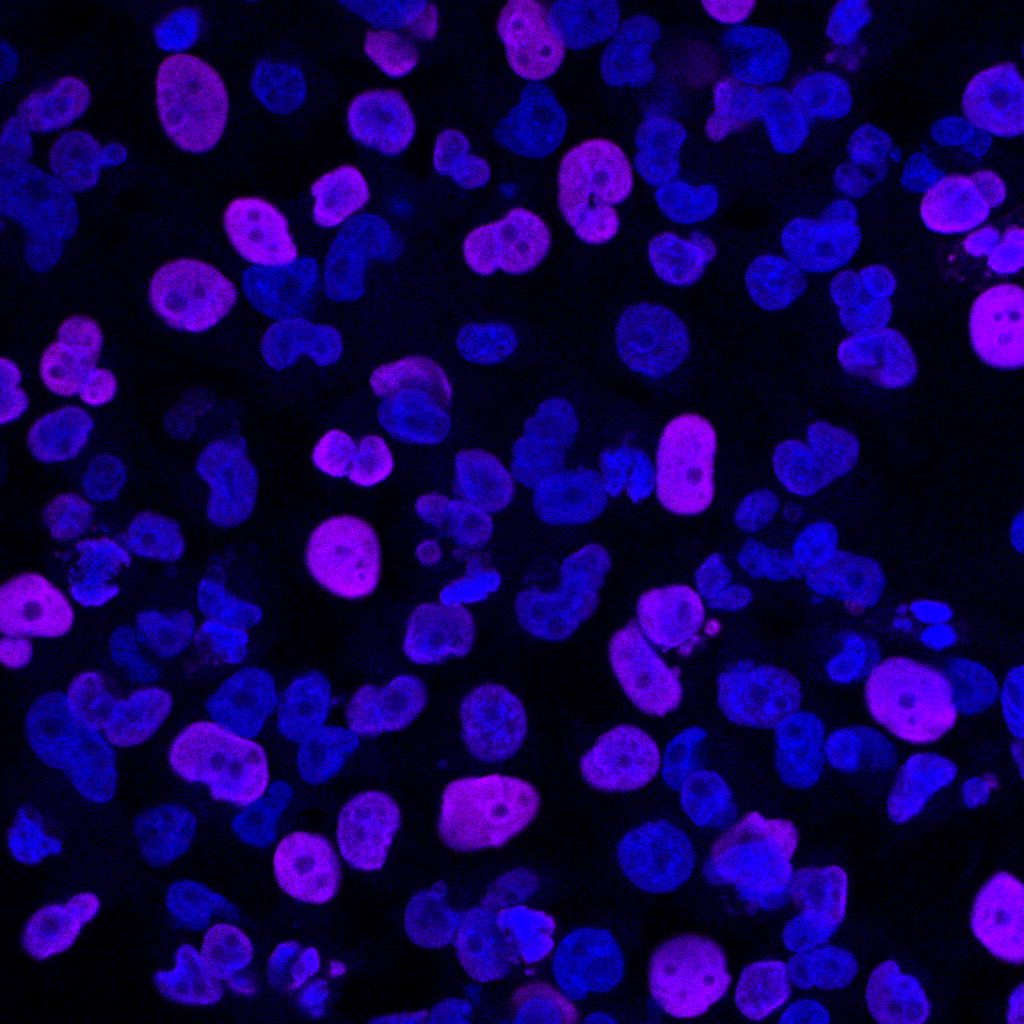

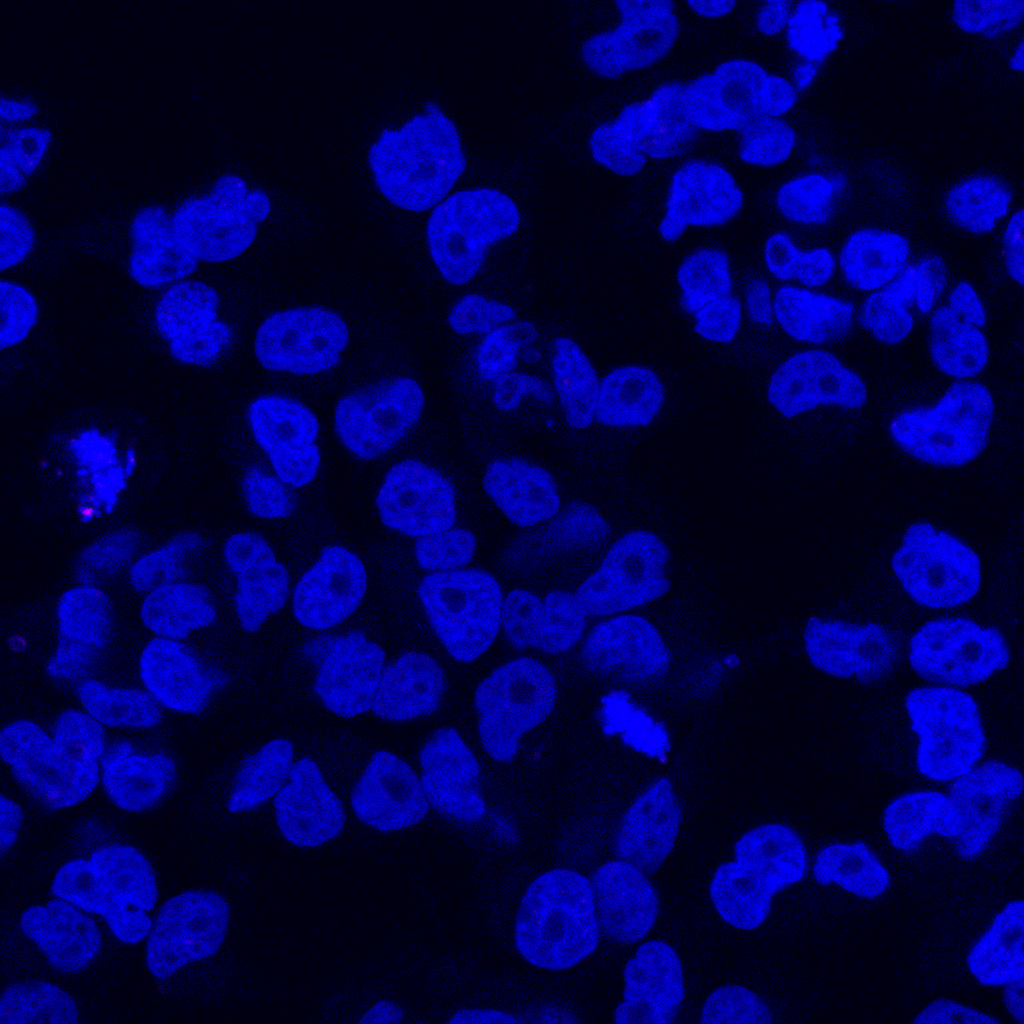

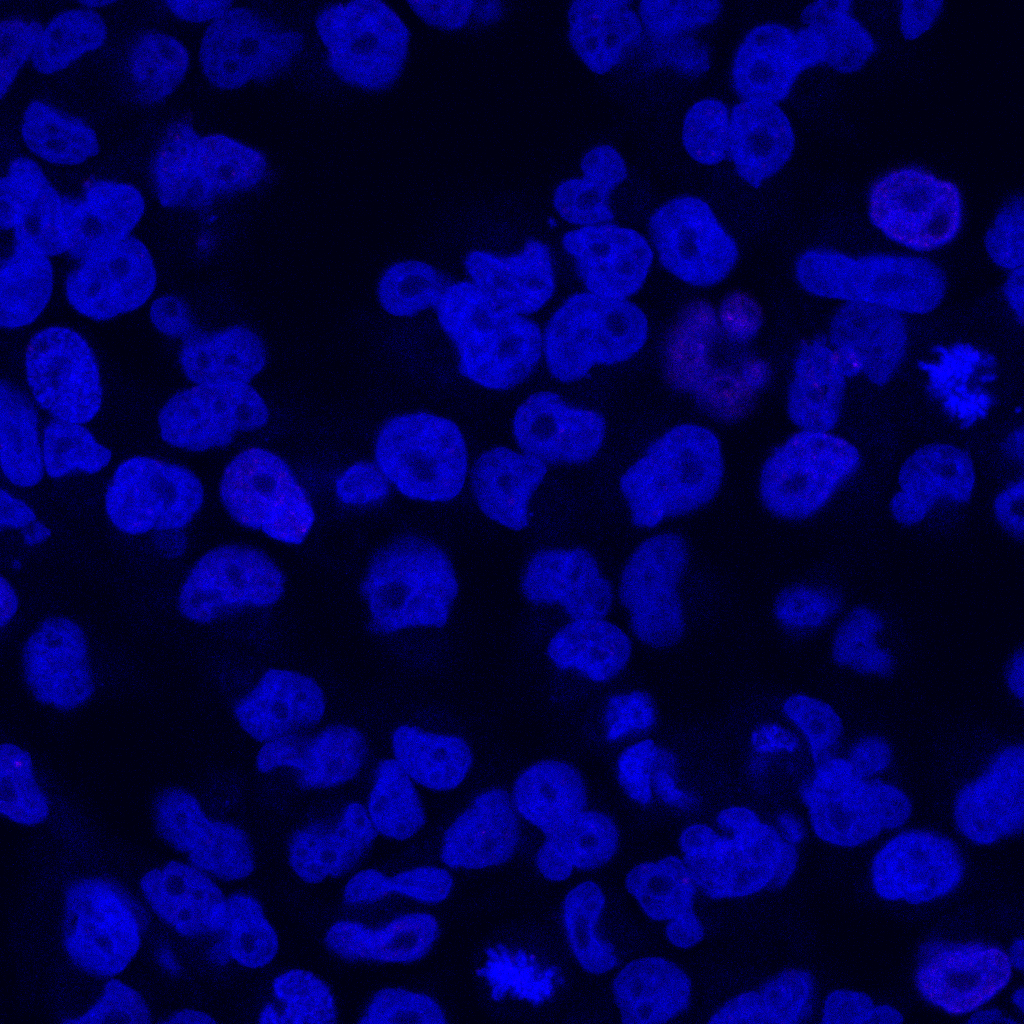

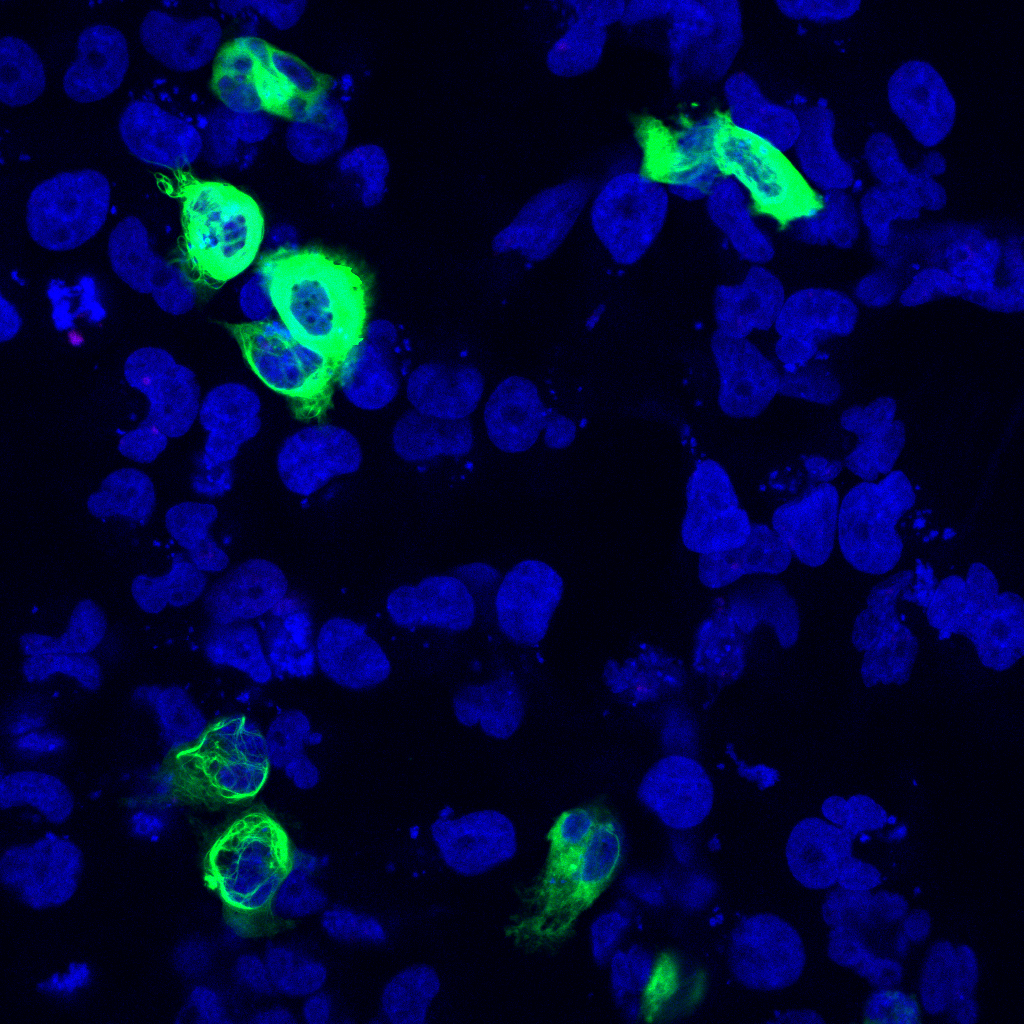

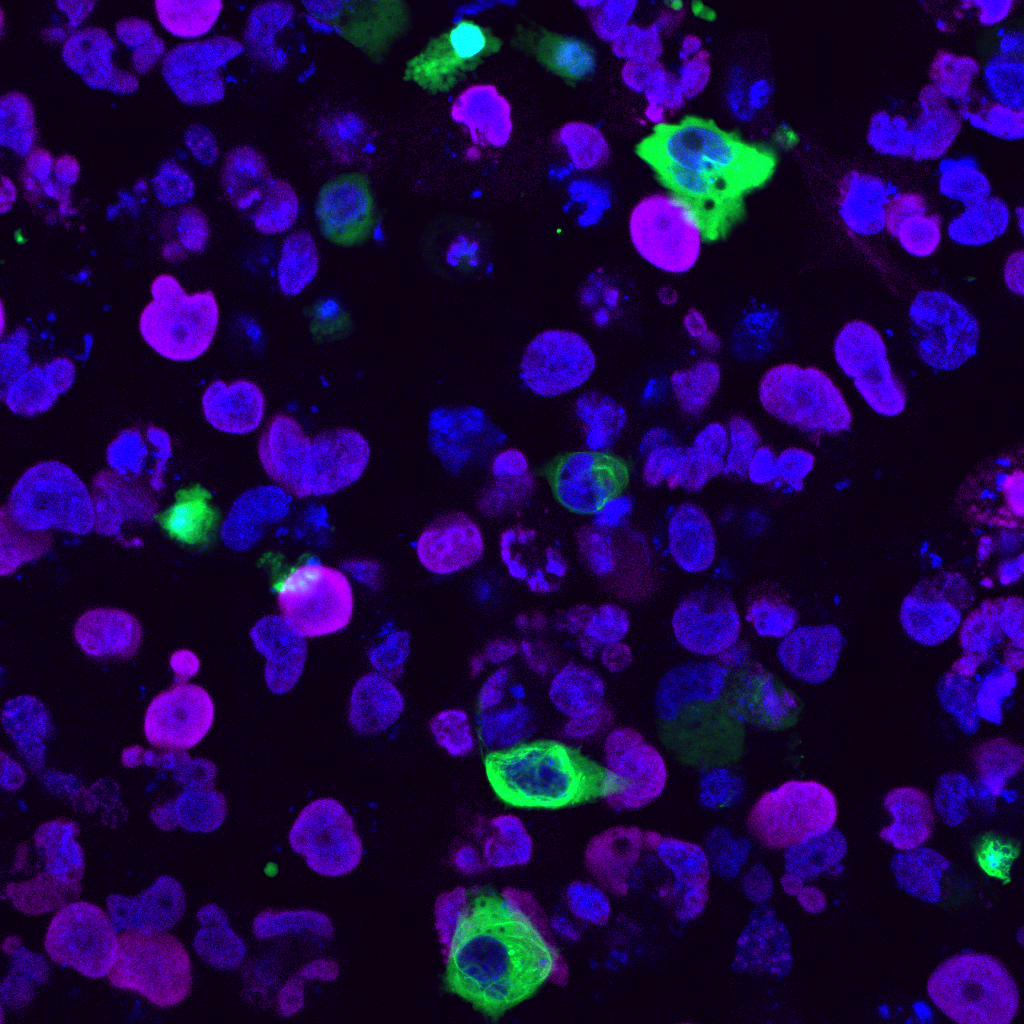

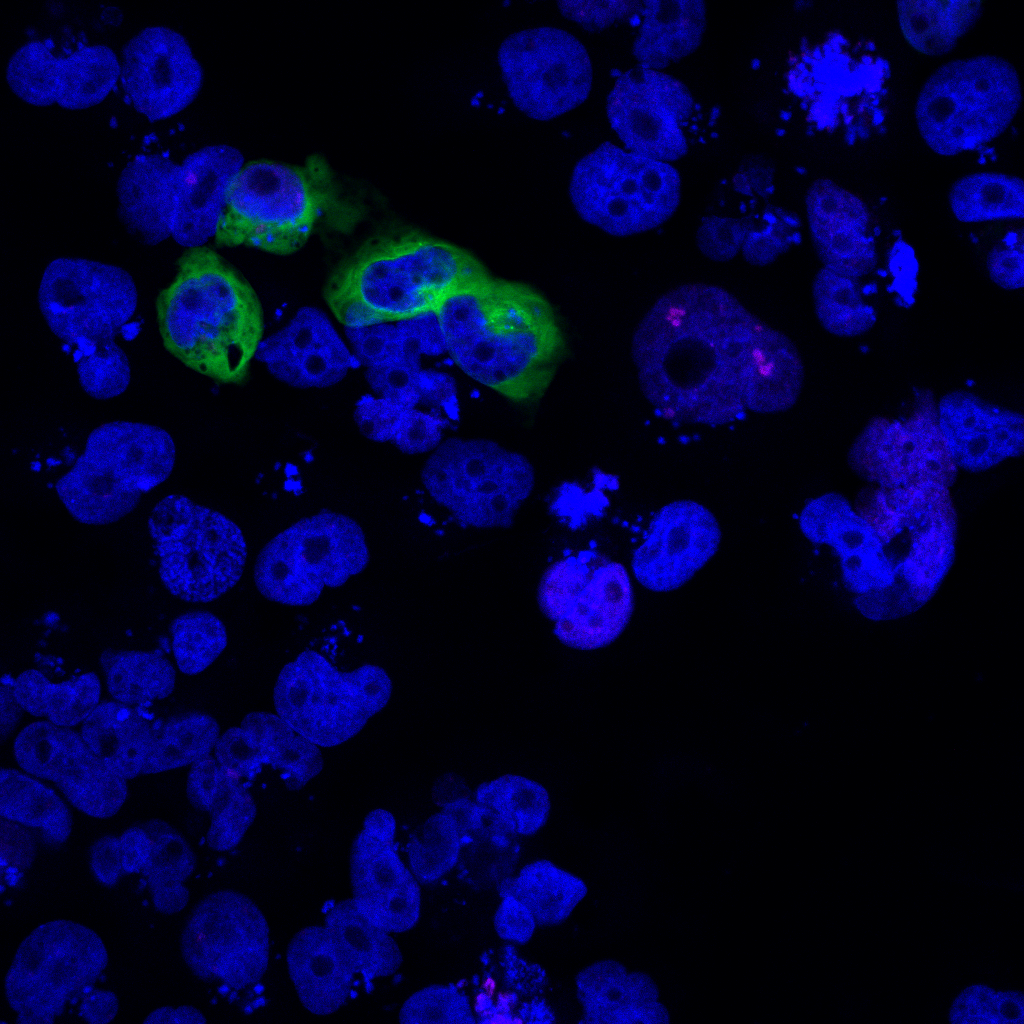

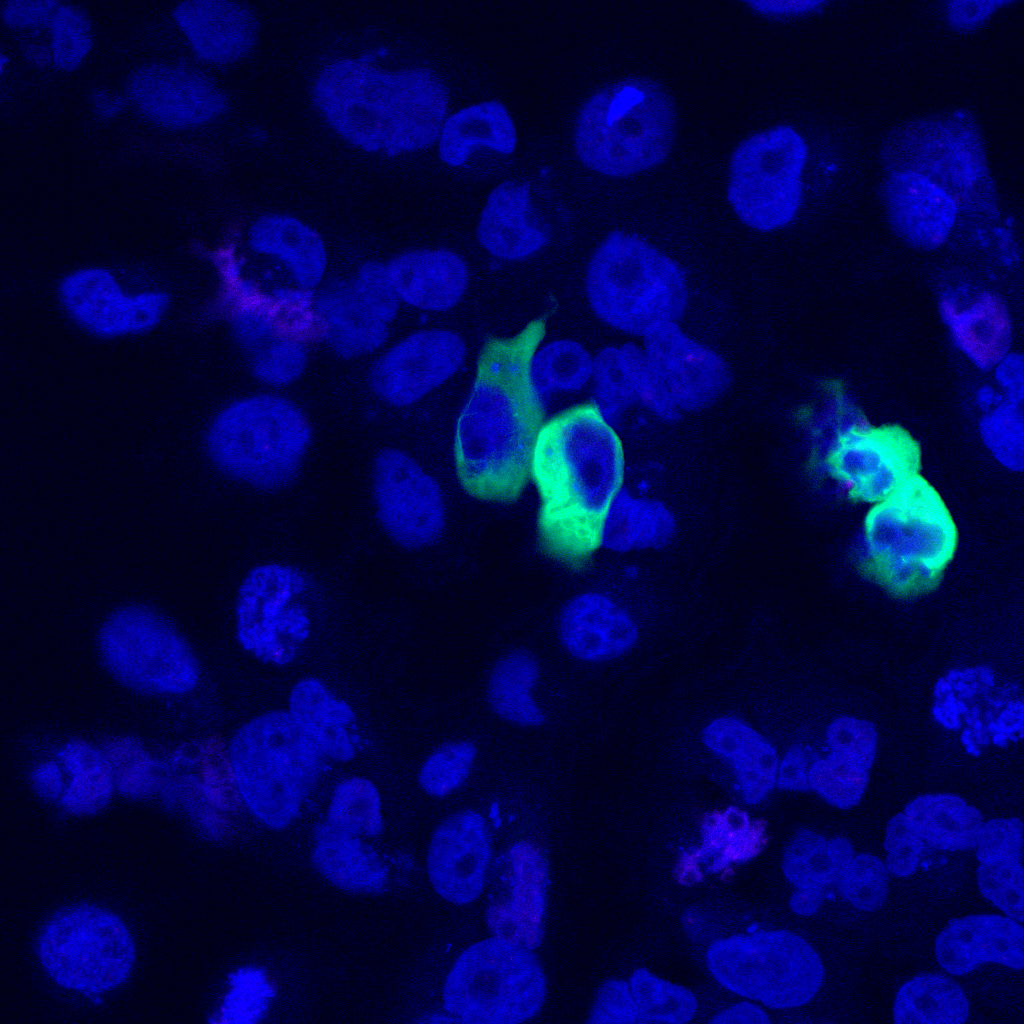


**pCCNB1**

**OVCAR8**

**OVCAR8R**

**Untreated**

**Prex**

**E**

**Supplementary Figure 3.**

**A**

**Control**

**OVCAR5**

**OVCAR5R**

**OVCAR8**

**OVCAR8R**

**p-HH1 (T154)**

**HH1**


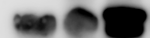

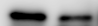

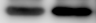

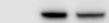


**p-HH1/total HH1**

**OVCAR5**

**OVCAR5R**

**OVCAR8**

**OVCAR8R**

**Prex (hr)**

**0 6 24 0 6 24**

**0 6 24 0 6 24**


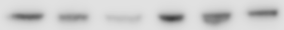


**pCDK1-Y15**


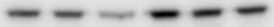

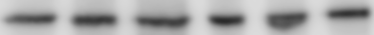

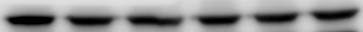


**GAPDH**


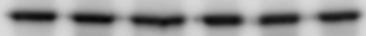


***pCDK1/CDK1***

**CDK1**


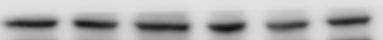


**1.0 0.7 0.3 1.0 0.9 0.8**

**1.0 1.0 0.5 1.0 0.9 0.9**

**B**

**Supplementary Figure 4.**

**A**


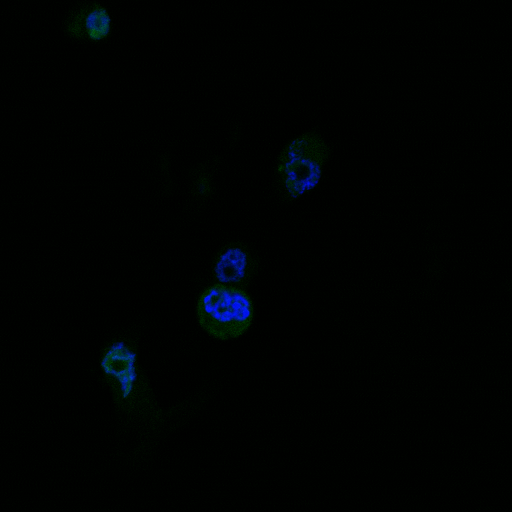

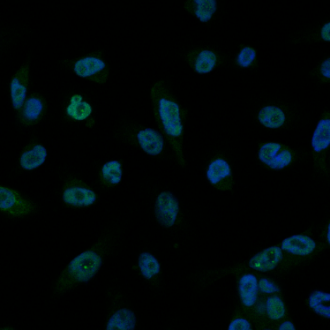

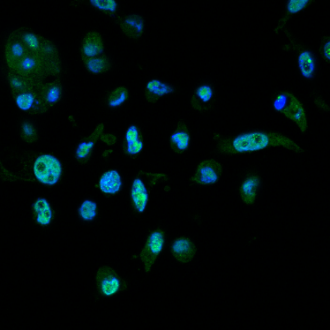

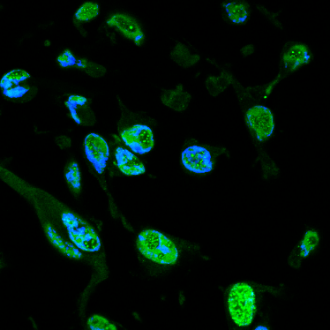

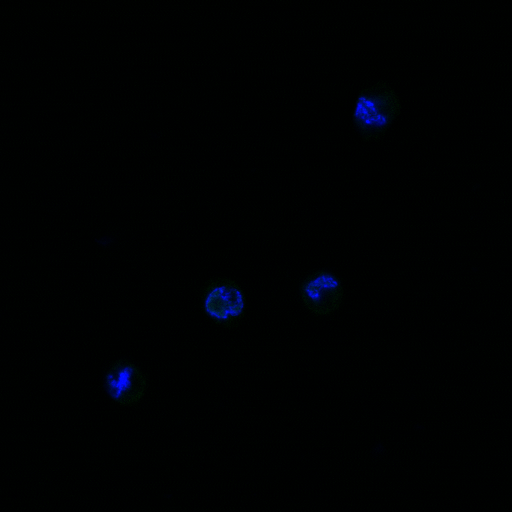

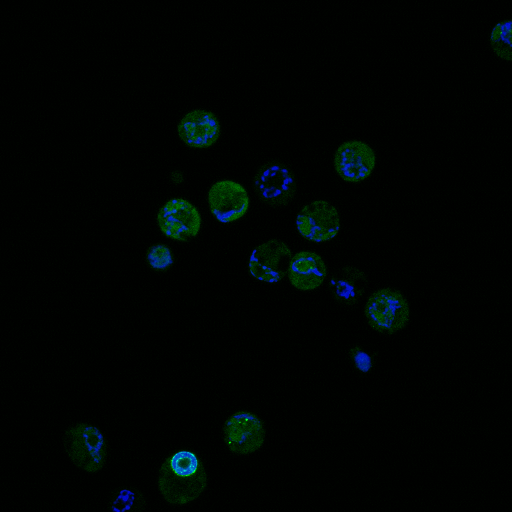

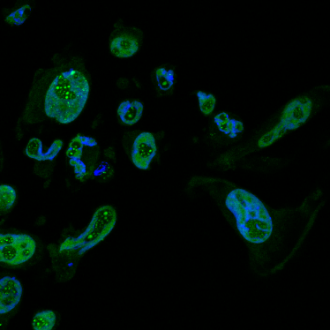


**OVCAR5**

**Untreated**

**Prex**

**Untreated**

**Prex**

**OVCAR8**


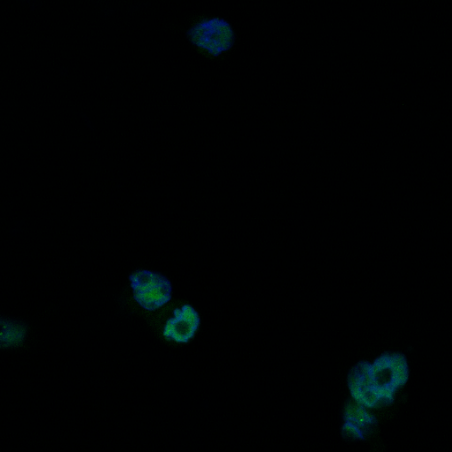


**OVCAR5R**

**OVCAR8R**

**DAPI; RPA70**


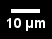

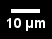


**10 µm**

**10 µm**

**B**

*******

*******

**RPA70 (FI)**

**OVCAR5**

**OVCAR5R**

**OVCAR8**

**OVCAR8R**

*******

**NS**

*******

*******

**Supplementary Figure 5.**


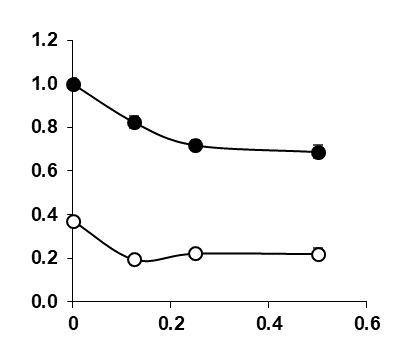

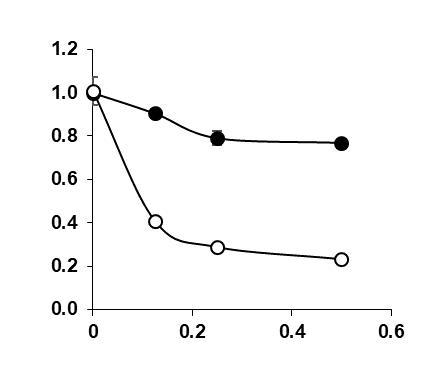

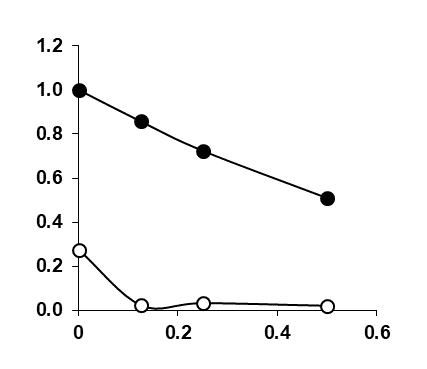

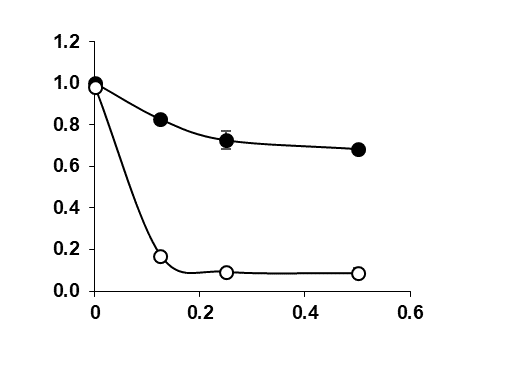


**Relative growth**

**OVCAR5**

**OVCAR5R**

**OVCAR8**

**OVCAR8R**

**Relative growth**

**HU (mM)**

**HU (mM)**

**HU (mM)**

**HU (mM)**

**Relative growth**

**Relative growth**

Untreated

Prex

Untreated

Prex

Untreated

Prex

Untreated

Prex

**A**

**B**

**REFERENCES**

1 Lee JM, Nair J, Zimmer A, Lipkowitz S, Annunziata CM, Merino MJ *et al*. Prexasertib, a cell cycle checkpoint kinase 1 and 2 inhibitor, in BRCA wild-type recurrent high-grade serous ovarian cancer: a first-in-class proof-of-concept phase 2 study. *Lancet Oncol* 2018; 19: 207-215.

2 Berglind H, Pawitan Y, Kato S, Ishioka C, Soussi T. Analysis of p53 mutation status in human cancer cell lines: a paradigm for cell line cross-contamination. *Cancer Biol Ther* 2008; 7: 699-708.

3 Reich M, Liefeld T, Gould J, Lerner J, Tamayo P, Mesirov JP. GenePattern 2.0. *Nature genetics* 2006; 38: 500-501.
